# Low‐Pt NiNC‐Supported PtNi Nanoalloy Oxygen Reduction Reaction Electrocatalysts—In Situ Tracking of the Atomic Alloying Process

**DOI:** 10.1002/anie.202203728

**Published:** 2022-07-27

**Authors:** Quanchen Feng, Xingli Wang, Malte Klingenhof, Marc Heggen, Peter Strasser

**Affiliations:** ^1^ The Electrochemical Energy Catalysis and Materials Science Laboratory Department of Chemistry Chemical and Materials Engineering Division Technische Universität Berlin 10623 Berlin Germany; ^2^ Ernst Ruska-Centre for Microscopy and Spectroscopy with Electrons Forschungszentrum Jülich GmbH 52425 Jülich Germany

**Keywords:** Atomic Alloying Process, Bimetallic Nanocatalyst, Oxygen Reduction Reaction, Polymer Electrolyte Membrane Fuel Cell, Structure–Activity Relationship

## Abstract

We report and analyze a synthetic strategy toward low‐Pt platinum‐nickel (Pt‐Ni) alloy nanoparticle (NP) cathode catalysts for the catalytic electroreduction of molecular oxygen to water. The synthesis involves the pyrolysis and leaching of Ni‐organic polymers, subsequent Pt NP deposition, followed by thermal alloying, resulting in single Ni atom site (NiNC)‐supported PtNi alloy NPs at low Pt weight loadings of only 3–5 wt %. Despite low Pt weight loading, the catalysts exhibit more favorable Pt‐mass activities compared to conventional 20–30 wt % benchmark PtNi catalysts. Using in situ microscopic techniques, we track and unravel the key stages of the PtNi alloy formation process directly at the atomic scale. Surprisingly, we find that carbon‐encapsulated metallic Ni@C structures, rather than NiN_*x*_ sites, act as the Ni source during alloy formation. Our materials concepts offer a pathway to further decrease the overall Pt content in hydrogen fuel cell cathodes.

## Graphical abstract

During thermal annealing treatment, carbon‐encapsulated Ni NPs, rather than NiN_*x*_ single sites, serve exclusively as the Ni atom source for PtNi alloy formation. Despite this low Pt weight loading, the catalysts exhibit more favorable Pt‐mass activities compared to conventional, carbon‐supported 20–30 wt %_Pt_ Pt‐loaded benchmark PtNi alloy catalysts.

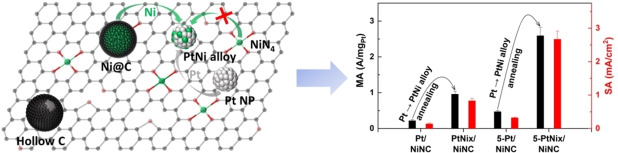

## Introduction

Hydrogen‐fueled polymer electrolyte membrane fuel cells (PEMFC) that generate electricity through the electrochemical oxidation of hydrogen, are an important component in the suite of emerging green and sustainable technology solutions to the looming global energy crisis.^[1]^ The kinetically sluggish oxygen reduction reaction (ORR) at PEMFC cathodes, however, continues to limit the overall PEMFC performance, causes large kinetic overpotentials, and still requires unacceptably high Pt loadings in the cathode catalyst layer.[^1a^, ^2^] Therefore, enormous research efforts have been put into the development of low‐Pt content cathode electrocatalysts and layers thereof. This has resulted in a number of new active and low‐Pt content cathode catalyst design concepts that meet and, in some cases, even exceed current technological Pt ORR mass activity targets.[^1e^, ^1g^, ^1i^, ^3^]

Among these catalyst design concepts, there is a hybrid catalyst design that combines platinum‐group metal (PGM)‐free transition metal (M)‐ and nitrogen‐doped carbon (MNC) material—an alternative to conventional carbon supports—together with Pt‐based nanoparticles (NPs). Following this concept, the drawbacks inherent in both MNC and Pt could be reciprocally offset.

On one hand, the presence of Pt could help to solve the critical stability issues encountered by MNC materials with single M‐N_*x*_ active sites. Jaouen et al. improved the stability of FeNC material through the incorporation of minute amounts (1–2 wt %) of Pt into FeNC.^[4]^ Subjecting this Pt/FeNC hybrid catalyst to reductive annealing treatment led to the formation of Pt@FeO_*x*_ core–shell structure, which resulted from the migration of some Fe from FeNC support to Pt surface. While this ORR‐inactive Pt@FeO_*x*_ structure did not directly contribute to the overall ORR activity, it still stabilized the FeNC catalyst by suppressing the formation of by‐product H_2_O_2_ and reactive oxygen species, which were known to be responsible for the fast degradation of FeNC material under fuel cell operations.

On the other hand, from the standpoint of Pt, the atomic proximity of single M‐N_*x*_ sites improved the performance of the hybrid Pt/MNC catalyst due to a number of reasons: first, theoretical calculations indicated that compared to undoped pure graphene, there existed a tighter binding and shorter distance between MNC and Pt surface.[^3b^, ^5^] This not only enhanced the interaction between Pt and MNC support, mitigating the agglomeration or detachment of Pt nanoparticles during operating conditions, but also lowered the barrier for charge transfer and hydrogen peroxide migration from MNC to Pt, thus facilitating the synergistic catalysis. Besides, FeN_4_ sites close to Pt could weaken the binding energy of O* on Pt(111) by 0.15 eV, approaching to the volcano peak (0.20 eV), therefore improving the intrinsic activity of Pt.^[5]^ Furthermore, densely and uniformly distributed ORR‐active single MN_4_ (mostly M=Fe and Co) sites on the surface of the MNC support could contribute to the overall apparent activity through two parallel reaction pathways, two‐ and four‐electron process.[^3b^, ^6^] Besides the direct contribution through four‐electron reaction pathway, in two‐electron reaction pathway, one so‐called tandem catalysis mechanism, that is incomplete conversion of O_2_ into H_2_O_2_ and subsequent migration of H_2_O_2_ into adjacent Pt sites, is proposed.[^3b^, ^3g^]

Due to the weak O* adsorption energy on single Ni site,^[6]^ ORR‐inactive NiNC catalysts, compared to their Fe and Co counterparts, have had a lower priority for the hybrid ORR catalyst design concept. However, NiNC supports offer other unexpected advantages that were previously overlooked. Despite its intrinsically poor ORR activity, NiNC provide other important benefits, including Fenton‐inactive character and homogeneous distribution of ionomer benefiting from its N‐doped feature^[1f]^ (see Note S4 and 5 for more details). On the other hand, although surface NiN_*x*_ site motifs are not ORR‐active, alloying Pt NPs with Ni into PtNi nanoalloys has resulted in the most reactive and highest‐performing PEMFC ORR cathode catalysts to date.[^1i^, ^7^] This bears the important and to‐date untested hypothesis whether NiNC catalysts with its highly dispersed Ni atoms both in bulk and on surface may offer unique advantages in terms of the supply of individual Ni atoms during the formation of ORR‐active and highly dispersed PtNi alloy NPs. In fact, other than NiN_*x*_ sites, metallic Ni nanoparticle agglomerates are often present in the bulk of NiNC catalysts due to high‐temperature pyrolysis involved during the preparation of NiNC catalysts. Thus, hypothetically, thermally treating a hybrid Pt/NiNC catalyst, consisting of Pt NPs supported on NiNC support, we may be able to trigger the Pt‐Ni particle alloying process, such that the Ni atoms are homogeneously supplied by either NiN_*x*_ sites or Ni NPs inside the catalyst itself. Compared to conventional Ni impregnation/reduction pathways towards nanoalloy particles, the distribution of the initial Ni atom source would be much more homogeneous. The viability of this unusual PtNi alloy NP formation process, and the unambiguous identification of the Ni atom source has never been considered before. This present study aims to change that by using in situ transmission electron microscopy (TEM) technique to unravel the atomic process during the formation of the PtNi nanoalloy particles from pristine Pt/NiNC hybrid material and demonstrating the formation of highly active ORR electrocatalysts.

**Figure 1:**
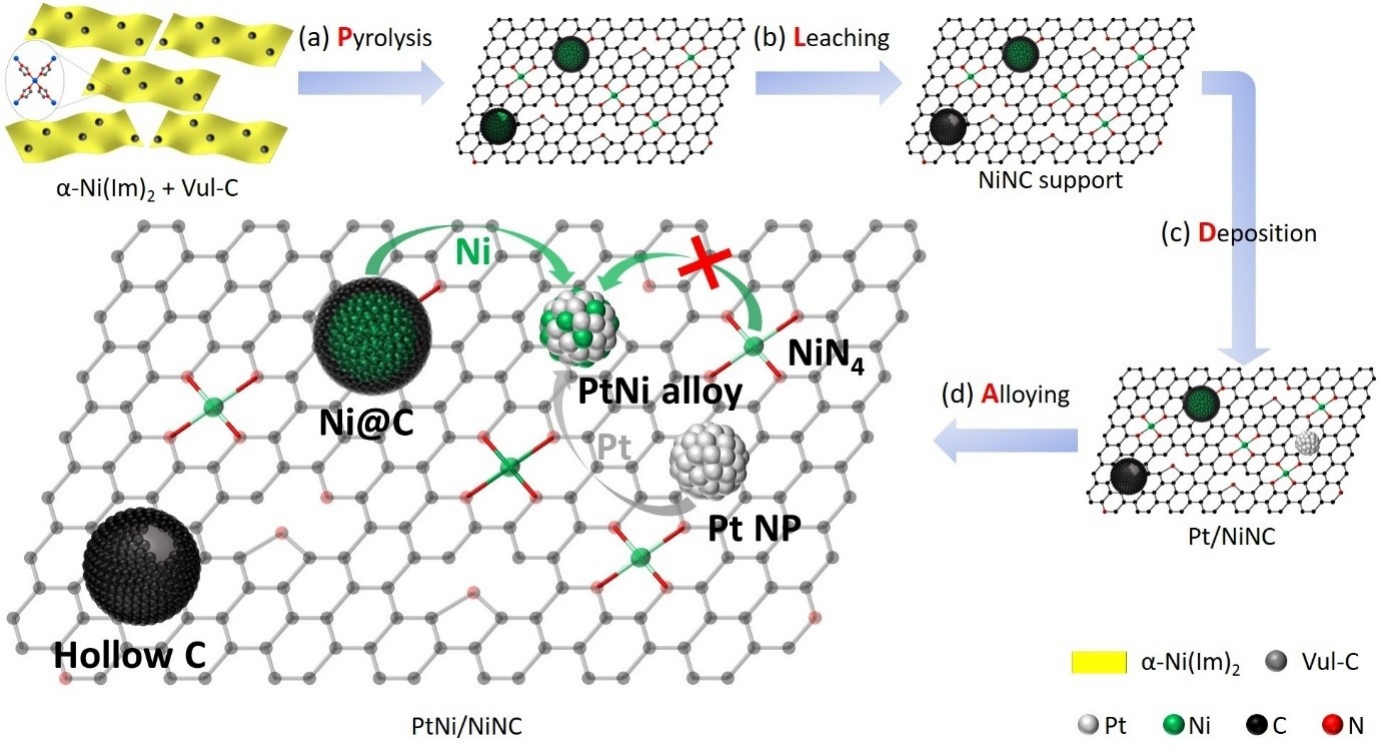
Schematic illustration of the pyrolysis‐leaching‐deposition‐alloying (PLDA) strategy reported in this study. a) Pyrolysis of homogeneous mixture of nickel coordination polymer (NCP) precursor, α‐Ni(Im)_2_, and Vulcan carbon. During high‐temperature pyrolysis, Ni cations in α‐Ni(Im)_2_ either were reduced to metallic Ni NPs, or were transformed to single NiN_*x*_ sites. b) Subsequent acid leaching to remove the partial unstable Ni NPs which were incompletely protected by the carbon shell, leaving a hollow carbon structure with an opening. Due to their acidic resistance, Ni NPs thoroughly covered by carbon shell, that was Ni@C structure, and single NiN_*x*_ sites were left unchanged in this corrosive step. c) In situ deposition of 3 wt % Pt NPs onto NiNC support via simple polyol process. d) Formation of PtNi alloy NP was triggered by thermal annealing treatment. In the enlarged inset, the green and grey arrows represented the supplying routes of Ni and Pt during alloying process, respectively, whereas one green arrow with red cross indicated that the supplying route of Ni from single NiN_*x*_ sites was not viable.

In this study, we investigate, validate and provide atomic insight into the hypothesized new synthetic pathway of ultralow PGM‐loaded PtNi alloy NPs supported on NiNC material (referred to as a four‐step PLDA process, see Figure 1). Using in situ heating TEM and electrochemical CO ad/desorption techniques, we demonstrate that the highly dispersed metallic Ni NPs, rather than single NiN_*x*_ sites, act as the supplying entity of individual Ni atoms for the formation of catalytically active, low‐Pt loaded (3–5 wt %), and uniform sized PtNi NPs. Subsequent electrochemical measurements via thin film rotating disk electrode (TF‐RDE) method show that our resultant PtNi/NiNC catalysts, in spite of their low Pt‐loaded (3–5 wt %) feature, exhibit comparable or even higher ORR mass activities than C‐supported, 20–30 wt % Pt‐loaded benchmark PtNi alloy catalysts, which would show great promise for future use in PEMFC cathodes.

## Results and Discussion

### Synthetic Design of Catalyst

Figure 1 and Figures S1, 2 illustrate the overall synthetic strategy of low‐Pt PtNi alloy NPs supported on Ni‐ and N‐doped carbon (NiNC) support. First, NiNC support was prepared via pyrolysis of a nickel coordination polymer (NCP) precursor [nickel(II) bisimidazolate, α‐Ni(Im)_2_]^[8]^ and subsequent acid leaching to remove partial metallic Ni particles (Steps a and b in Figure 1). Then Pt NPs were deposited onto NiNC support at very low weight loading (≈3 wt %) via facile polyol process, resulting in the pristine Pt/NiNC hybrid catalyst (Step c in Figure 1). To form the final active low‐Pt PtNi nanoalloy catalyst, the pristine Pt/NiNC hybrid catalyst was subjected to a second annealing treatment in 4 % H_2_/Ar (Step d in Figure 1).

### Sustainable Synthesis of Low‐Pt PtNi/NiNC Catalysts

As demonstrated in Figure S2e, our present PEMFC cathode catalyst synthetic method was easily scalable and was able to supply reproducible catalyst material at gram‐scale from one single batch synthesis (see Note S1 for more details). We note that the present synthesis involves inexpensive metal precursors, such as metal nitrates and chlorides, rather than expensive, unstable and toxic organometallic compounds, such as metal acetylacetonates or carbonyls. Direct and in situ deposition of Pt NPs onto support material was achieved via simple and facile polyol process.[^1f^, ^9^] Unlike many other liquid‐phase synthetic approaches generally carried out in either hydrophobic [oleylamine (OAm), octadecene (ODE), oleic acid (OA) or benzyl ether] or hydrophilic [dimethyl formamide (DMF)] solvents, the current ethylene glycol (EG) polyol route is rather sustainable and “green”.^[9b]^ This mainly results from two reasons: first, the coordinating/chelating properties of polar EG molecule are sufficient to functionalize and stabilize Pt NPs, which avoid the use of additional capping agent, and therefore circumvent the inherently complicated removal of surfactant molecules (OAm, OA or PVP) from the surface of Pt NPs before practical catalytic application. The weak interaction between EG and Pt NP and water solubility of EG allowed a simple water wash to easily clean the Pt NP surface. Second, the reducing power of EG at elevated temperatures makes additional reducing agent dispensable.

### Powder X‐ray Diffraction (XRD) and X‐ray Photoelectron Spectroscopy (XPS) Analysis

**Figure 2:**
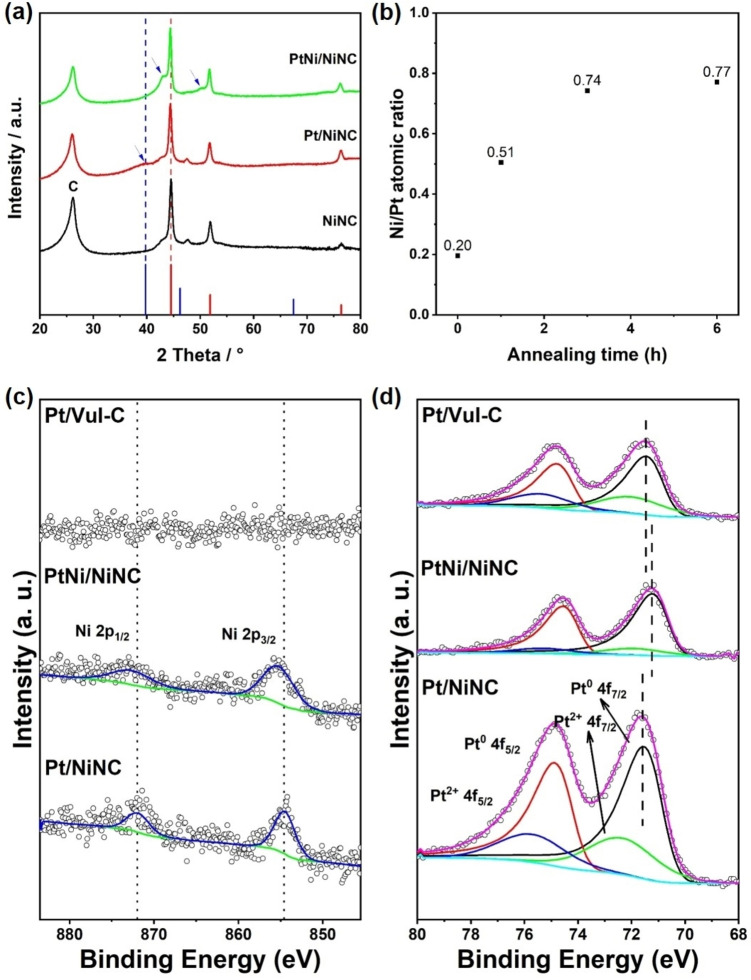
Chemical and structural characterizations of catalyst materials derived from Pt/NiNC before and after annealing treatment. a) XRD patterns of NiNC support, pristine Pt/NiNC and annealed PtNi/NiNC materials. Blue arrows indicate the shift of Pt peaks before and after annealing treatment. Standard patterns of Pt (blue lines, PDF No. 00‐004‐0802) and Ni (red lines, PDF No. 99‐000‐2639) are also shown. b) Change of Ni/Pt atomic ratio versus the annealing time in hours. By normalizing the total integral areas of Ni 2p and Pt 4f regions to their corresponding relative sensitivity factors (RSFs), the Ni/Pt atomic ratios were obtained from XPS spectra. c) Ni 2p and d) Pt 4f XPS spectra of pristine Pt/NiNC and annealed PtNi_*x*_/NiNC, together with Pt/Vul‐C reference material. XPS fitting was carried out by using CASA XPS software. For Pt^0^ species, asymmetric line shape of LA (1.2, 85, 70) was used, whereas for Pt^2+^ species, line shape of GL (30) was chosen. These vertical dashed lines are present as a guide for eyes.

XRD patterns in Figure 2a reveal structural characteristics of the synthetic intermediates: after pyrolysis and acid leaching, NiNC support demonstrates highly graphitized carbon as well as metallic Ni phases. This NiNC material served as support for the deposition of Pt NPs with ≈3 wt % loading. The Pt/NiNC hybrid material featured a characteristic broad Pt(111) Bragg reflection, marked by blue arrow in Figure 2a. After further annealing treatment applied to induce the PtNi alloy formation, the (111) diffraction peak moved in between those of monometallic Pt and Ni. According to Vegard's linear rule applied to the lattice parameter of face‐centered cubic (fcc) PtNi solid solution alloy (Figure S3),^[10]^ the shift of the (111) reflection toward higher 2‐Theta degree implied a decrease in the cubic unit cell parameter, indicating the successful incorporation of Ni atoms into the Pt NP lattices, forming PtNi alloy NPs. This observation was further confirmed by X‐ray photoelectron spectroscopy (XPS) results (see more details in Figures S4–S7 and Tables S1–S3). By normalizing the total integral areas of Ni 2p and Pt 4f regions to their corresponding relative sensitivity factors (RSFs), we could obtain the Ni/Pt atomic ratio from XPS spectra (Figure 2b, Figure S7 and Tables S3). With increasing the annealing time, the XPS‐derived experimental Ni/Pt atomic ratio increased from initial 0.2 in the pristine non‐annealed Pt/NiNC and gradually up to 0.77 in the final PtNi/NiNC alloy catalyst (with an annealing time of 6 h). Given the conservation of Ni and Pt in the overall sample, this appears counterintuitive, however, can be rationalized based on the supply of Ni metal atoms from XPS‐invisible metallic Ni NPs encapsulated by thick carbon layers. To show this, we also prepared Ni‐free Vulcan carbon‐supported Pt NP catalyst (referred to as Pt/Vul‐C) using the identical procedure as for Pt/NiNC. The comparison with Pt/Vul‐C allowed the unambiguous assignment of Ni 2p core level peaks of Pt/NiNC to N‐coordinated single Ni atomic sites on the surface (Figure 2c). The experimental increase in Ni/Pt atomic ratio during and after the thermal annealing of the Pt/NiNC suggested that Ni species diffused across the carbon encapsulation and participated in the PtNi alloying process. XPS spectra in Figure 2d showed that upon annealing treatment, Pt 4f peaks shifted to lower binding energy, indicative of charge transfer from Ni to Pt due to the formation of the binary alloy phase.[^2c^, ^11^] Hence, we hypothesize that the Ni atoms in the final PtNi alloy NPs derive to a large portion from the Ni NPs present in the NiNC support. To test our hypothesis, we use in situ heating TEM technique, which permits investigation of the evolution of our catalyst material during heating treatment.^[12]^

### In Situ Thermal TEM Analysis

**Figure 3:**
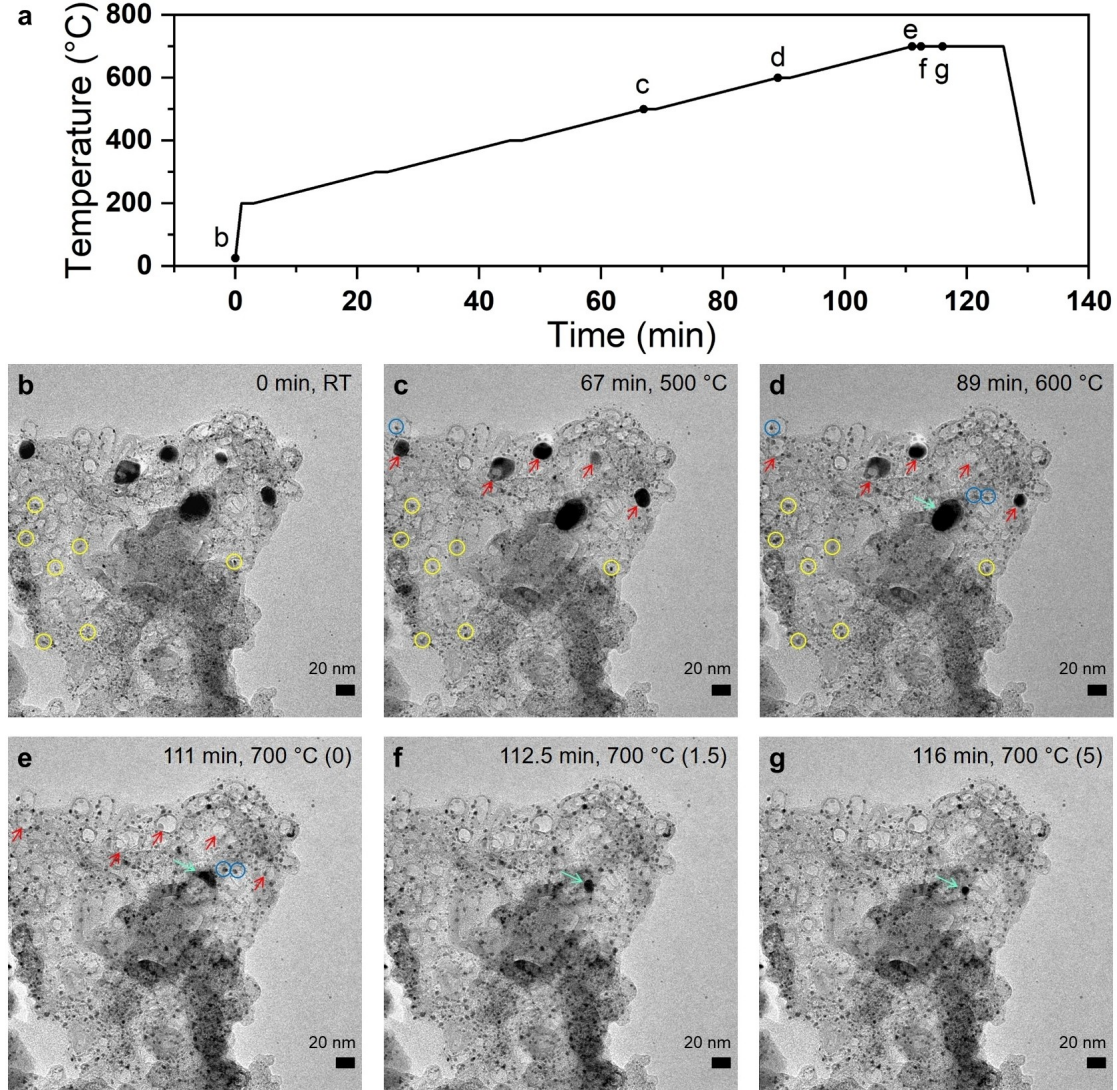
Investigation of the evolution from Pt to PtNi alloy using an in situ heating TEM technique. In situ TEM imaging of the pristine hybrid Pt/NiNC catalyst during heating from 25 °C (RT) to 700 °C under vacuum condition. a) Temperature profile over time with marked points corresponding to the images in panels (b–g). Starting from 200 °C, temperature was increased in step of 100 °C (5 K/min). The specimen was annealed for 2 min at each step below 700 °C and 15 min at final 700 °C. Yellow circles in panels (b–d) mark the Pt NPs agglomerated. Red arrows in panels (c–e) mark the evolution of Ni NPs. Light green arrows in panels (d–g) mark the same Ni NPs. In panels (e–g), the numbers in parentheses indicate the holding time in minutes at 700 °C.

A customized in situ heating TEM chip setup enabled us to design experiments to directly track the structural and morphological changes of the Pt/NiNC catalyst during thermal annealing (see Figure 3 and Video S1). We selected one specific field of view, in which smaller Pt and larger Ni NPs coexist, and simultaneously followed their morphological evolution during a stepwise thermal annealing from 25 °C to 700 °C (Figure 3a). Thanks to their varying particle size, visual distinction between Pt and Ni NPs was obvious. Starting from 200 °C, the temperature was held constant for 3 min at 100 °C increments, whereas it was held constant for 15 min at the final temperature of 700 °C. Up to 500 °C, compared with the initial state (Figure 3b), no significant changes in morphology were observed (Figure 3c). Only the smallest observable Pt NPs (yellow circles in Figures 3b, c and Figure S8) showed slight agglomeration or rounding. The critical temperature threshold, where Ni NPs started to change their morphology drastically was between 500 and 600 °C. At 600 °C, the TEM analysis revealed that individual Ni NPs shrank in size and gradually vanished. Figure 3d shows five Ni NPs marked by red arrows. Numbered from left to right, the first and fourth Ni NPs disappeared, whereas the others shrank. This observation suggested that Ni atoms started to diffuse across the carbon bulk to nearby regions and adjacent Pt NPs. The mobile Ni atoms were incorporated into Pt NPs to yield bimetallic PtNi alloy NPs. Direct evidence of this process is provided in Figures 3d–g (light green arrows), where one Ni NP first shrank in size before it performed a random walk to coalesce with nearby Pt NPs into a larger bimetallic PtNi alloy NP. Further holding at 700 °C up to 15 min did not lead to any further changes (Figure S9). Besides, careful reference experiments were carried out to ensure the absence of any beam damage effect. Details on these experiments and conclusions are provided in the Note S2.

Additional evidence of the key role of the large Ni NPs as Ni atom source for the formation of the PtNi alloy NPs was provided from separate ex situ STEM/EDX as well as in situ heating TEM experiment (see Note S3 and Figures S11–S19), in which a Ni NP‐free NiNC material (1 wt % of Ni, thus referred to as “NiNC‐1”) served as catalyst support. Deposition of Pt NPs resulted in the Pt/NiNC‐1 catalyst material. STEM‐EDX analysis of Pt/NiNC‐1 material (Figures S14 and S15) showed carbon‐embedded single Ni atom sites next to small Pt NPs before and after thermal annealing. In situ heating TEM studies analogous to those mentioned above confirmed both the absence of Ni NPs and the thermal stability of the supported Pt NPs (Figure S16 and Video S2). There was no experimental evidence for the formation of bimetallic PtNi alloy NPs. Thus, we conclude that the N‐coordinated singe Ni atom sites, NiN_*x*_, embedded in the carbon matrix of the NiNC‐1 support were not released to form bimetallic alloys with the deposited Pt NPs.

### Surface Voltammetry and CO‐Stripping

**Figure 4:**
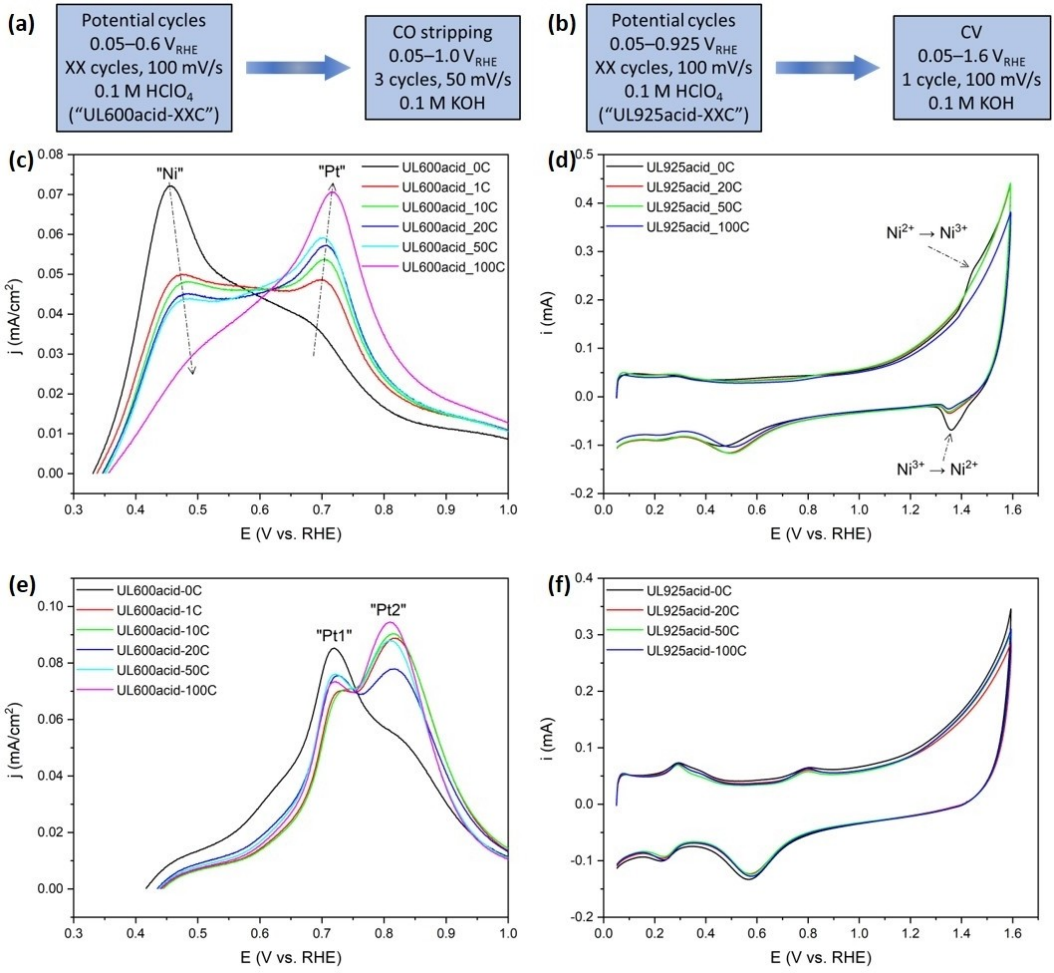
Surface characterization by CO electrooxidation and CV measurements in alkaline electrolyte. (Left) Electrochemical pre‐treatments in 0.1 M HClO_4_ acidic electrolyte and (right) subsequent measurements in 0.1 M KOH alkaline electrolyte for a) CO electrooxidation and b) CV measurements. c, e) Pt surface area‐normalized and background‐subtracted CO stripping voltammetry and d, f) CV curves in 0.1 M KOH solution for a series of catalyst films undergoing pretreatment in acidic solution with different potential cycling numbers: c, d) annealed PtNi/NiNC and e, f) pristine non‐annealed Pt/NiNC samples, respectively.

To verify the successful formation of the final bimetallic PtNi alloy NP from the individual carbon‐encapsulated Ni NP upon thermal annealing, we carried out voltammetric surface CO stripping (electrooxidation to CO_2_) as well as cyclic voltammetry (CV) to rather anodic potential of 1.6 V_RHE_ (RHE, reversible hydrogen electrode), both in alkaline electrolyte. CO stripping voltammetry in alkaline solutions is one of the most surface‐sensitive techniques to probe the presence of Ni atoms in or near the surface of Pt alloy catalysts.^[13]^ Prior to CO stripping in alkaline solution, the powder catalyst films were pretreated in acidic solution and thoroughly rinsed thereafter. Two potential cycling pretreatment protocols (Figures 4a, b) with distinct upper potential limit (UL) were applied. The pretreatment UL prior to CO stripping tests was set to 0.6 V_RHE_, whereas the UL of CV tests was 0.925 V_RHE_. The reason why we employ a more cathodic UL for CO stripping was to prevent the formation of Pt oxides, which would affect the evolution of surface composition.^[14]^ Lower CO mobility in alkaline electrolyte than in acidic electrolyte results in multiple peaks, which result from the contributions of Pt surface sites with different activity toward CO oxidation.[^14^, ^15^] This offers us the ability to distinguish the Pt surface sites under different chemical environment.^[16]^ Figure 4c shows background‐subtracted CO stripping voltammograms of a series of annealed PtNi/NiNC catalysts after acidic pretreatment involving varying numbers of potential cycles. The current was normalized to the real Pt surface area obtained from the CO stripping peak area. The voltammetric CO stripping in Figure 4c involves two redox waves: first, a wave at lower potential, labelled “Ni”, at 0.4–0.5 V_RHE_ that corresponds to electrooxidation of CO on Pt sites in proximity to (sub)surface Ni sites, and another stripping peak at higher potential, labelled “Pt”, at around 0.7 V_RHE_ corresponding to CO oxidation at Pt site ensembles. Monotonic trends in “Pt” and “Ni” peak areas evidenced a gradual surface Ni dissolution with increasing acidic cycle numbers: the “Ni” CO stripping charge decreased, whereas the “Pt” CO stripping charge increased. CV scans of a series of annealed PtNi/NiNC catalysts in 0.1 M KOH (Figure 4d) exhibited the characteristic Ni^2+^/Ni^3+^ redox waves suggesting the presence of Ni atoms in the alloy NPs.

By contrast, when increasing the cycle numbers during acidic pretreatment of the pristine non‐annealed Pt/NiNC material, there was no discernible “Ni” CO stripping peak nor a Ni redox wave in the CV (Figures 4e, f), evidencing absence of Ni atoms on the Pt surface. In Figure 4e, the pristine non‐annealed Pt/NiNC material showed the familiar main CO oxidation wave at 0.7 V_RHE_ (labelled as “Pt1”), followed by a second one at around 0.8 V_RHE_ (labelled as “Pt2”). From these voltammetric results, we conclude that the thermal treatment of the Pt/NiNC precursor material resulted in bimetallic PtNi alloy phases in the final PtNi/NiNC catalyst.

To provide further independent evidence that encapsulated Ni NPs serve exclusively as the Ni atom source for the formation of PtNi alloy NP, CO stripping and surface voltammetry were carried out using the Ni NP‐free reference catalyst material, Pt/NiNC‐1, as mentioned earlier (see Figures S11–S19 for more details of synthesis and characterization, and Note S3 for conclusions). These results in alkaline electrolyte (Figures S19a, b) revealed the absence of both “Ni”‐type CO oxidation waves and Ni^2+^/Ni^3+^ redox peaks, respectively. Hence, the Pt NPs after thermal annealing remained Ni‐free. From this, we conclude that N‐coordinated single Ni atom centers, NiN_*x*_, are unable to supply Ni atoms to form the PtNi alloy phase, which confirms that metallic carbon‐encapsulated Ni NPs must act as the main source of Ni species in the process of alloy formation.

Besides, to demonstrate the general applicability of our PLDA synthetic strategy to generate active PtNi alloy catalyst even at somewhat higher than 3 wt % Pt loading (Figures 2, 3 and 4), we then prepare a 5 wt % Pt‐loaded catalyst materials (Figures S20–S22), herein denoted as 5‐Pt/NiNC and 5‐PtNi/NiNC, respectively.

### Electrocatalytic Oxygen Reduction Reaction (ORR) Activity

**Figure 5:**
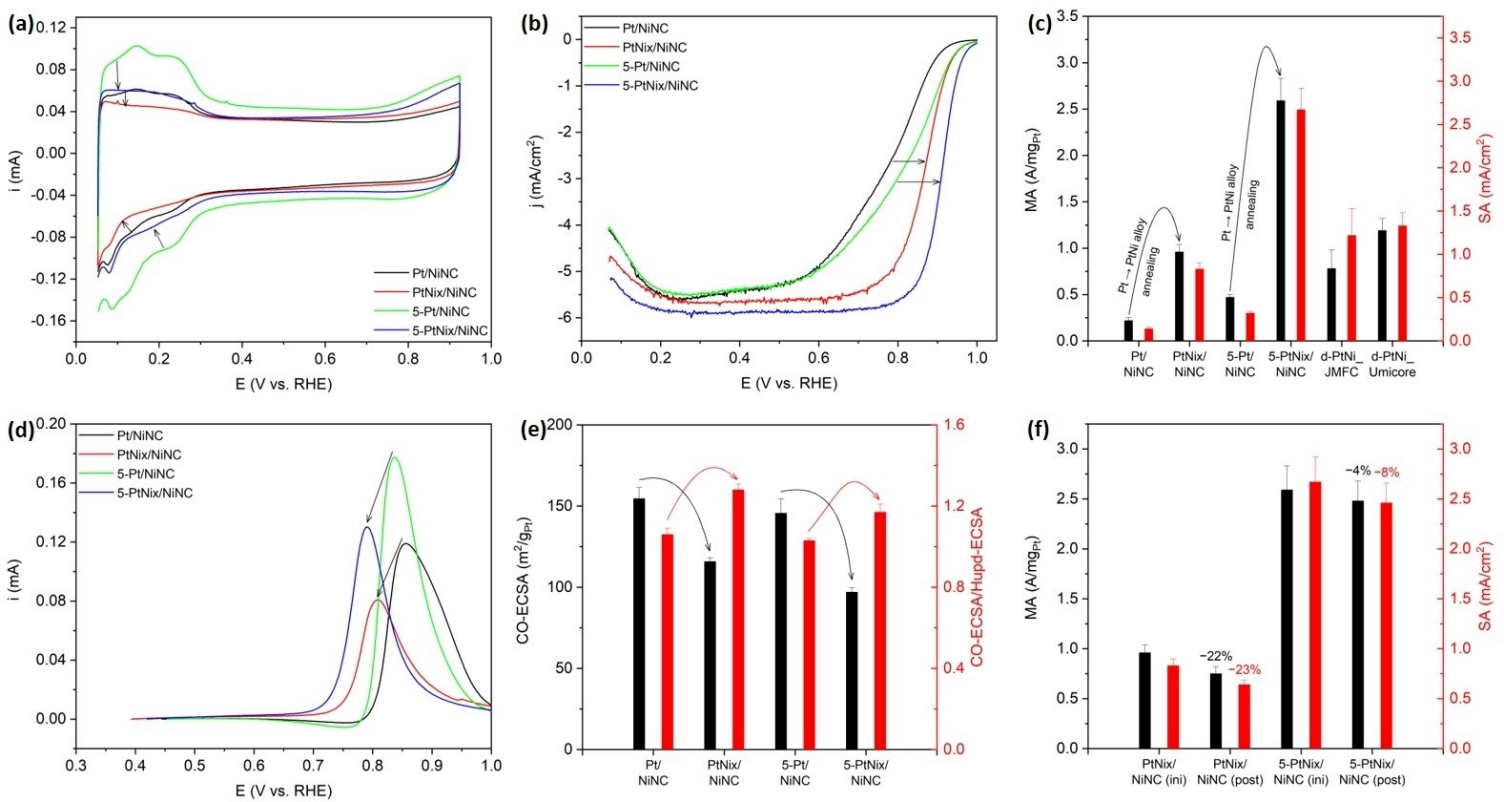
RDE electrochemical measurements on Pt/NiNC‐based catalyst samples with two dealloyed PtNi alloy benchmark catalysts. a) Cyclic voltammetry (CV) curves of pristine Pt/NiNC and annealed PtNi/NiNC catalyst samples. Black arrows indicate the decrease of H_upd_ regions. b) ORR polarization curves. Black arrows indicate the right shift of half‐wave potential. c) Comparison of mass activities (MA) and specific activities (SA) measured at 0.9 V_RHE_. d) Background‐subtracted CO stripping voltammetry curves. Black arrows indicate the cathodic shift of the CO oxidation peak after annealing treatment. e) CO‐ECSA values estimated from CO stripping method and the ratios of CO‐ECSA versus H_upd_‐ECSA. Black curved arrows indicate the decrease of CO‐ECSA values, whereas red ones indicate the increase of the ratio of CO‐ECSA/H_upd_‐ECSA. f) Comparison of MA and SA before and after the accelerated durability test (ADT), which is 10 000 CV cycles between 0.6 and 0.925 V_RHE_ at 100 mV s^−1^. Geometric Pt mass loading: 1.9 μg cm^−2^ for Pt/NiNC, 1.9 μg cm^−2^ for PtNi/NiNC, 3.2 μg cm^−2^ for 5‐Pt/NiNC, 3.2 μg cm^−2^ for 5‐PtNi/NiNC, 14.7 μg cm^−2^ for d‐PtNi_JMFC, and 14.9 μg cm^−2^ for d‐PtNi_Umicore.

To evaluate the electrocatalytic ORR reactivity of the PtNi/NiNC electrocatalysts, we performed thin film‐rotating disk electrode (TF‐RDE) experiments (Figure 5).^[17]^ Figure 5a shows the initial cyclic voltammograms (CV) in N_2_‐saturated electrolyte. As indicated by black arrows, after annealing, both H_upd_ adsorption and desorption regions become smaller. This results from a NP growth along with the formation of PtNi alloy NPs with (sub)surface Ni atoms, which is known to partially suppress H_upd_ surface adsorption.[^1i^, ^3a^, ^18^] To evaluate the catalytic ORR reactivity, linear sweep voltammetry (LSV) in O_2_‐saturated electrolyte was conducted. The LSV polarization curves in Figure 5b showed a kinetic improvement in ORR activity over the pristine non‐annealed catalysts. Pt mass activities (MA) and specific activities (SA) were calculated by using the Koutecky–Levich formalism and subsequent normalization by Pt mass loadings and Pt surface area, respectively. As seen in Figure 5c, the MA and SA values of PtNi/NiNC catalyst at 0.9 V_RHE_ exhibit improvement factors of 4.4× and 5.9×, respectively, compared with pristine Pt/NiNC catalyst. And for 5‐PtNi/NiNC catalyst, its MA and SA values increased improved by 5.5‐ and 7.2‐fold, respectively. For comparison with commercial PtNi ORR fuel cell electrocatalysts, two dealloyed PtNi benchmark catalysts supplied from Johnson Matthey Fuel Cell (JMFC) and Umicore (Figures S23 and S24) were also measured.

In the evaluation of the intrinsic specific ORR activity, that is, the reactivity based on the real surface area, care had to be taken: as seen in Figure 5a, due to the relatively low content of Pt in these catalyst materials, combined with the partially suppressed sorption of H_upd_ on PtNi alloy surface, the H_upd_ sorption features between 0.05 and 0.4 V_RHE_ are expectedly very weak.[^3a^, ^18^] This, however, can lead to a large experimental error in the evaluation of the H_upd_ charge (Q_H_), the ECSA, and ultimately an inaccurate overestimation of intrinsic ORR activities.^[19]^ Thus, we employed CO stripping voltammetry, rather than H_upd_ method in this report to calculate the real Pt surface area. Background‐subtracted CO stripping curves in Figure 5d shows that after annealing treatment, on one hand, the Pt surface area drops due to the agglomeration of Pt NPs, on the other hand, the CO oxidation peak shifts toward lower potential, indicating that the formation of PtNi alloy phase modifies the electronic structure of Pt and promotes the CO electrooxidation performance.[^1a^, ^2c^, ^16^, ^19^, ^20^]

Transformation from Pt into PtNi alloy phase is further confirmed by the increase of the CO‐ECSA/H_upd_‐ECSA ratio after annealing treatment, as marked by the red curved arrows in Figure 5e. Despite the difficulty in determining the accurate H_upd_ charge due to low Pt loading, it is still interesting that the ratio of CO‐ECSA versus H_upd_‐ECSA could provide indicative information about the alloy formation.[^1i^, ^3a^, ^18^] For the sake of consistency and better comparison between different samples, the H_upd_ integral area was obtained by integrating the current in the same potential range (0.05–0.4 V_RHE_).

Stability tests were performed by cycling the potential between 0.6 and 0.925 V_RHE_ for 10 000 potential cycles at a sweeping rate of 100 mV s^−1^. As shown in Figure 5f, after 10 000 cycles, the MA and SA of PtNi/NiNC catalyst show activity losses of 22 % and 23 %, respectively. For 5 wt % Pt‐loaded counterpart, MA and SA of 5‐PtNi/NiNC catalyst, display much smaller activity losses of 4 % and 8 %, respectively.

## Conclusion

In this contribution, we presented a novel PLDA synthetic pathway toward highly ORR active NiNC‐supported PtNi alloy nanocatalysts and demonstrated the scalability of this preparation route to the gram‐scale. The key novelty lies in the leverage of C‐encapsulated metallic Ni (Ni@C) NPs—present in the bulk of NiNC support material—as Ni atom supply for the formation of the PtNi alloy NPs. The high initial dispersion of the Ni NP, which is inherited from Ni‐containing coordination polymer precursor, allows for a homogeneous supply of individual Ni atoms across the material for the PtNi alloy formation at the Pt NP sites. For the first time, we visually tracked the concomitant PtNi alloy particle formation and the disappearance of Ni@C NPs at the atomic scale using in situ heating TEM experiments. We observed how the Ni NPs shrank while the catalytic PtNi NPs grew on the support. To verify our microscopic findings, we also used electrochemical CO ad/desorption experiments to sensitively probe the presence of Ni atoms at the (sub)surface of the PtNi NPs. Using carefully chosen reference experiments, we demonstrated that the NiN_*x*_ single sites did not supply any Ni atoms thanks for their compositional stability. RDE measurements showed that our highly active PtNi alloy electrocatalysts, albeit their 3–5 wt % low‐Pt loading, achieved comparable or even higher mass activities than conventional, C‐supported, 20–30 wt % Pt‐loaded benchmark PtNi alloy catalysts. More generally, in addition to these Pt‐based catalysts investigated in this work, we believe that the validity and generality of our new PLDA approach would allow us to prepare other noble metal‐based bimetallic NPs for other energy‐related electrochemical reactions.

## Experimental Section

The experimental details about the synthesis of precursor materials, preparation of pristine and annealed hybrid catalyst materials, characterization techniques, electrochemical measurements in both acidic and alkaline electrolytes are provided in Supporting Information.

## Conflict of interest

The authors declare no conflict of interest.

1

## Supporting information

As a service to our authors and readers, this journal provides supporting information supplied by the authors. Such materials are peer reviewed and may be re‐organized for online delivery, but are not copy‐edited or typeset. Technical support issues arising from supporting information (other than missing files) should be addressed to the authors.

Supporting Information

Supporting Information

Supporting Information

## Data Availability

The data that support the findings of this study are available in the supplementary material of this article.
