# Supplementary material for: Low‐Pt NiNC‐Supported PtNi Nanoalloy Oxygen Reduction Reaction Electrocatalysts—In Situ Tracking of the Atomic Alloying Process
Source: Angew Chem Int Ed Engl. 2022 Jul 27;61(36):e202203728. doi: 10.1002/anie.202203728 (PMC9544639; doi:10.1002/anie.202203728)
Supplement: Supplementary file 1 — Supporting Information [file ANIE-61-0-s002.pdf]

Angewandte Chemie
Eine Zeitschrift der Gesellschaft Deutscher Chemiker

Supporting Information

Low-Pt NiNC-Supported PtNi Nanoalloy Oxygen Reduction Reaction Electrocatalysts—In Situ Tracking of the Atomic Alloying Process

Q. Feng, X. Wang, M. Klingenhof, M. Heggen, P. Strasser**

Table of Contents

Table of Contents 2
Experimental Procedures 3
Catalyst preparation 3
Catalyst characterizations 3
Electrochemical measurements 4
Supporting Figures, Supporting Tables and Supporting Notes 6
Supporting Videos 41
Video S1 41
Video S2 41
References 42
Author Contributions 43

Experimental Procedures

Catalyst preparation

Synthesis of α-Ni(Im)$_2$ precursor: Typically, $Ni(NO_3)_2 \cdot 6H_2O$ (30 mmol, 8.724 g) was dissolved in 600 mL deionized H_2O. Then, solid imidazole (24 g, 352.5 mmol) was added to this aqueous solution. NaOH aqueous solution (0.3 M, 1200 mL) was added dropwise at a rate of 60 mL min^{-1}. The flow rate was controlled by a peristaltic pump. The resulting suspension was filtered and washed with water and ethanol several times and dried in a heating oven overnight at 70 °C. The as-obtained yellow powder was α-Ni(Im)$_2$ precursor.[1]

Synthesis of NiNC support material: 550 mg of Vulcan carbon (XC72R) and 5.5 g of α-Ni(Im)$_2$ were poured into a beaker which contained 1 L of water and 20 mL ethanol. The above suspension was vigorously stirred for 30 min, followed by sonication in a water bath for another 30 min. This agitation-sonication process was cycled for several times until Vulcan carbon and α-Ni(Im)$_2$ were thoroughly and homogeneously mixed together. The solid mixture was filtered and dried in a freeze dryer. Pyrolysis of α-Ni(Im)$_2$ was directly carried out under a nitrogen flow at 900 °C for 3 h. The ramping rate from RT to 900 °C is 5 °C min^{-1}. The pyrolyzed sample was then soaked in excess dilute sulfuric acid (H_2SO_4) solution (0.2 M) at 70 °C for 12 h to remove the partially exposed Ni NPs. Then the product was centrifugated and washed with copious water and dried in a freeze dryer.

Preparation of the pristine Pt/NiNC catalysts: Herein, the as-obtained NiNC was used as support material for the deposition of ultralow amounts of Pt NPs. The functionalization of NiNC by Pt NPs was carried out via simple polyol process.[2] 1800 mg of NiNC was added into a 1000 mL round bottom flask which contained 300 mL of ethylene glycol and 150 mL of DI water. To obtained a homogeneous solution, the above suspension was sonicated in a water bath for 15 min. Then 1854 µL of H_2PtCl_6 solution (0.192 M) was added into the above solution and stirred vigorously at room temperature for 18 h. To complete the reduction of the Pt precursor, the reaction flask was immersed in a 120 °C pre-heated oil bath and stirred under reflux condition for 2 h. The product was collected by centrifugation and washed with water/ethanol mixture for several times and dried in a freeze dryer. The actual Pt content was measured by ICP-OES.

For 5 wt% Pt-loaded catalyst, 3159 µL of H_2PtCl_6 precursor solution (0.192 M) was added while other parameters were kept same.

Preparation of PtNi/NiNC catalysts: The as-synthesized Pt/NiNC catalysts were annealed under 4% H_2/Ar at 700 °C for 6 h. And the ramping rate from RT to 700 °C is 5 °C min^{-1}.

Synthesis of NiNC-1 support material: NiNC-1 support material was synthesized by following a previously described method[3] and the synthetic procedure was illustrated in **Figure S12**. Precursor mixture consisting of $Ni(Ac)_2 \cdot 4H_2O$ (23.2 mg), 1,10-phenanthroline (phen, 200 mg) and ZIF-8 (800 mg, Basolite Z1200 from BASF) was ball-milled at 400 rpm for 1 h (3 × 20 min with 15 min rest in-between) in a ZrO_2 crucible (45 cm^3) filled with 100 ZrO_2 balls (5 mm diameter). This mixture was then pyrolyzed in nitrogen at 1000 °C for 1 h. The catalyst powder was collected and gently ground with a marble mortar and pestle without any post-treatment. The resultant Ni content is 1.2 wt%, and Zn content is 3.6 wt%.

Catalyst characterizations

X-ray powder diffraction (XRD) patterns were collected using a D8 Advance Diffractometer (Bruker) equipped with a Lynx Eye Detector and KFL Cu 2K X-ray tube. The measurement was carried out at a step size of 0.04°, in a 2θ range between 20 and 80°.

The metal contents of the different catalysts were determined by inductively coupled plasma mass spectroscopy (ICP-MS) using a 715-ES-ICP analysis system (Varian). The samples were prepared by dissolving the catalysts powders in a mixture of 2 mL H_2SO_4, 2 mL HNO_3 and 6 mL HCl. The solutions were heated from RT to 180 °C in 10 min using a Microwave Discover SP-D (CEM corporation), and the temperature was held at 180 °C for 20 min. After cooling down to RT, the solutions were filtered and diluted with ultrapure water to a known volume (50 mL). To estimate the concentration of the solution, 4 standard solutions of Pt and Ni with a known concentration were prepared. The concentrations of Pt were 0, 5, 10 and 20 mg/L, whereas the concentrations of Ni were 0, 10, 20 and 40 mg/L. The selected wavelengths for Pt measurement were 203.646, 204.939, 214.424, 224.552 nm, 265.945 nm and 306.471 nm. The selected wavelengths for Ni measurement were 227.021 nm, 230.299 nm, 222.295 nm, 222.486 nm, 221.648 nm, 230.078 nm, 216.555 nm and 231.604 nm.

Transmission electron microscopy (TEM) images were recorded on a FEI Tecnai G^2 20 S-TWIN with a LaB_6 cathode operating with 200 kV acceleration voltage and a resolution limit of 0.24 nm. Samples were dispersed in ethanol with an ultrasonic horn (~15 min), drop casted on a Cu grid (400 mesh), and dried in air at 60 °C for 10 min.

X-ray photoelectron spectroscopy: XPS measurements were carried out in an ultrahigh vacuum (UHV) chamber at room temperature using a non-monochromatized X-ray source (Al Kα, 1486.6 eV) and a hemispherical analyzer (Phoibos 150, SPECS). The Casa XPS software was used for analyzing the obtained spectra. All spectra were aligned according to the C1s main peak which was assigned with a binding energy of 284.3 eV. The Ni/Pt atomic ratios were obtained from the area under the Ni 2p and Pt 4f peaks after normalization by their corresponding relative sensitivity factors (RSF) of 21.1 and 15.86, respectively.

High resolution TEM: HRTEM was performed using a FEI Titan 80-300 TEM with a Cs corrector for the objective lens (CEOS GmbH). The microscope was operated at 300 kV.

Scanning transmission electron microscopy (STEM) was performed using a FEI Titan 80-200 (“ChemiSTEM”) electron microscope with a Cs-probe corrector (CEOS GmbH) and an HAADF detector. The microscope was operated at 200 kV. In order to achieve “Z-Contrast” conditions, a probe semi-angle of 25 mrad and an inner collection semi-angle of the detector of 88 mrad were used. Compositional maps were obtained with energy-dispersive X-ray spectroscopy (EDX) using four large-solid-angle symmetrical Si drift detectors. For EDX elemental mapping, Pt L and Ni K peaks were used. The error of the EDX composition measurement for individual particles after a typical investigation of 15 minutes is about ±2 at.%.

***In situ* heating TEM** study was performed at a FEI Tecnai $G^2$20 S-TWIN transmission electron microscope with a LaB_6 cathode operated at an accelerating voltage of 200 kV (ZELMI Centrum, Technical University of Berlin). For in situ TEM experiments, a heating holder (DENS solutions B.V.) was applied. The catalyst powder was first dispersed into isopropanol solution with ultrasonication. Then this catalyst solution was drop cast onto a MEMS chip. The precise control of temperature was realized by four-point measurement integrated on the chip. After complete drying under ambient condition, the heating chip was mounted on a TEM holder.

Electrochemical measurements

5 mg catalyst was added to a solution of 3.98 mL ultrapure water, 10 µL 5 wt% Nafion ionomer solution and 1 mL isopropanol. The suspension was then sonicated with an ultrasonic horn sonifier for 30–40 min while immersed in an ice water bath. An aliquot of the ink was drop casted by a pipette onto a glassy carbon (GC) rotating disk electrode (RDE, diameter: 5 mm, geometrical surface area: 0.196 cm^2). The film was dried at 60 °C for 8 min.

For electrochemical characterizations a conventional three electrode cell with a graphite rod as counter electrode, a mercury/mercury(I) sulfate electrode (MMS, Hg/Hg_2SO_4) in a Luggin capillary as reference electrode and a catalyst-coated GC-RDE as working electrode was used. MMS was calibrated regularly by a homemade reversible hydrogen electrode (RHE) using a polycrystalline Pt disk and continuous hydrogen bubbling. All potentials are referred to RHE. Freshly prepared 0.1 M $HClO_4$ diluted from concentrated $HClO_4$ (99.999% trace metal bases, Sigma Aldrich) with ultrapure water was used as electrolyte. The catalyst-coated RDE was controlled by a rotator from Pine Research instrument. All measurements were performed with a BioLogic SP-200 potentiostat. Gases for purging of electrolyte were H_2 (99.999%), N_2 (99.999%) and O_2 (99.998%).

To avoid bubbles to be trapped on the dry catalyst film, it was thoroughly wetted until an uniform thin water film could emerged. Then the catalyst-coated RDE was immersed into the N_2-saturated electrolyte under potential control at 0.05 V_{RHE}. For conditioning of the catalyst film, 50 potential cycles from 0.05 to 0.925 V_{RHE} at 100 mV/s were carried out. 3 potential cycles from 0.05 to 0.925 V_{RHE} at 20 mV/s were performed subsequently.

Electrochemical impedance spectroscopy (EIS): the as-measured results were iR-corrected manually after measurements, where the value of R was determined by EIS technique. A potential of 0.5 V_{RHE} was applied for 1 min, followed by an impedance measurement from 100,000 Hz to 10 Hz with an AC amplitude of 10 mV. The resistance R was then determined from the Im (Z) vs. Re (Z) plot.

ORR testing: For background correction, measurements at 1600 rpm were first carried out in N_2-saturated electrolyte. A potential of 0.05 V_{RHE} was applied for at least 30 s. Then LSV was measured between 0.05 and 1.0 V_{RHE} at 20 mV/s and repeated once. The electrode was then raised above the electrolyte. After purging of pure oxygen through the electrolyte for 10 min, the electrode was reinserted under an applied potential of 0.05 V_{RHE}, and another three LSV measurements at 20 mV/s and one LSV measurement at 5 mV/s were conducted. In the case of a noticeable decrease in diffusion limited currents, oxygen was bubbled in between the scans.

Analysis of ORR activity: First the potentials were iR-corrected. Then capacitance correction was applied. LSV curve performed in N_2-saturated electrolyte was used as background and subtracted from the LSV curve performed in O_2-saturated electrolyte at the same scan rate. ORR current (i_{ORR}, at 0.9 V_{RHE}) and diffusion limiting current (i_L, at 0.4 V_{RHE}) were obtained from the iR- and capacitance-corrected LSV curve. Finally the kinetic current i_k was calculated by Koutecky-Levich equation. The mass activity (MA) was calculated by dividing i_k by the Pt mass loading on the electrode. And the specific activity (SA) was was calculated by dividing i_k by the Pt area estimated from CO stripping method.

Stability test (RDE protocol): The stability test was performed after carrying out the activation, impedance measurement and activity measurements. The rotation was turned off, the electrode was raised from the electrolyte under potential control and N_2 was bubbled for 10 min. Then the electrode was immersed and the potential was cycled between 0.6 and 0.925 V_{RHE} at 100 mV/s for 10,000 cycles, while venting above the electrolyte with N_2. After that the potential was cycled as described in “activation”, but only 3 cycles at 100 mV/s and 3 cycles at 20 mV/s. Then impedance spectroscopy and ORR activity test after stability test were also carried out.

CO stripping: initial CO stripping measurement was performed after initial ORR test. After ORR test, N_2 was bubbled for 10 min. The electrode was reinserted under an applied potential of 0.05 V_{RHE} and the rotation was set to 400 rpm. Then CO was bubbled for another 1 min at the same rotation speed. The gas was switched back to N_2 and the electrolyte was bubbled for another 10 min to

remove excess non-adsorbed CO in the electrolyte. Then the rotation was turned off and three CV cycles were recorded at 50 mV/s between 0.05 V_{RHE} and 1 V_{RHE}, while venting N_2 above the electrolyte.

Analysis of CO stripping: The CO stripping data were analyzed to calculate the CO-ECSA and the ratio of CO-ECSA/H_{upd}-ECSA. For the CO-ECSA, the positive scan of the second cycle was used as background and subtracted from the positive scan of the first cycle. Then the integral of the peak was calculated in the iR-corrected potential range from the crossing of the curves to the highest potential (~1 V_{RHE}). The integral area was normalized by the scan rate and the Pt mass loading on the electrode. A value of 420 µC/cm^2 was used for the electrooxidation of a monolayer of adsorbed CO. The H_{upd}-ECSA after CO stripping was calculated using the cycle after CO stripping. H_{upd}-ECSA was determined by integrating the current in the negative going sweep. Due to the low content of Pt, it is quite hard to determine the accurate potential range of H_{upd} region. For consistency and better comparison, we selected 0.4 V_{RHE} as the upper potential limit, whereas the lowest potential was chosen as lower potential limit (~0.05 V_{RHE}). Subtraction of the capacitive background current was applied. The integral area was normalized by the scan rate and the Pt mass loading on the electrode. A value of 210 µC/cm^2 was used for the adsorption of a hydrogen monolayer.

CO stripping in 0.1 M KOH alkaline electrolyte: for CO stripping in alkaline electrolyte, electrochemical pre-treatments in 0.1 M $HClO_4$ acidic electrolyte were first performed. The potential was cycled between 0.05 and 0.6 V_{RHE} at 100 mV/s in deaerated 0.1 M $HClO_4$ acidic electrolyte. Catalyst that underwent different potential cycles in acidic electrolyte were denoted as “UL600acid-XXC”. UL600 means that the upper potential limit during cycling is 600 mV_{RHE}, and XXC denotes the actual number of potential cycles. After electrochemical pre-treatment in acidic solution, the electrode was rinsed by ultrapure water. CO stripping was then performed following the same procedure as described in the section of “CO stripping”, except that 0.1 M KOH alkaline electrolyte was used.

CV in 0.1 M KOH alkaline electrolyte: for CV test in alkaline electrolyte, electrochemical pre-treatments in 0.1 M $HClO_4$ acidic electrolyte were first performed. The potential was cycled between 0.05 and 0.925 V_{RHE} at 100 mV/s in deaerated 0.1 M $HClO_4$ acidic electrolyte. Catalyst that underwent different potential cycles in acidic electrolyte were denoted as “UL925acid-XXC”. Herein, UL925 means that the upper potential limit during cycling is 925 mV_{RHE}, and XXC denotes the actual number of potential cycles. After electrochemical pre-treatment in acidic solution, the electrode was rinsed by ultrapure water. CV was performed in N_2-saturated 0.1 M KOH alkaline electrolyte between 0.05 and 1.6 V_{RHE} at 100 mV/s.

Supporting Figures, Supporting Tables and Supporting Notes

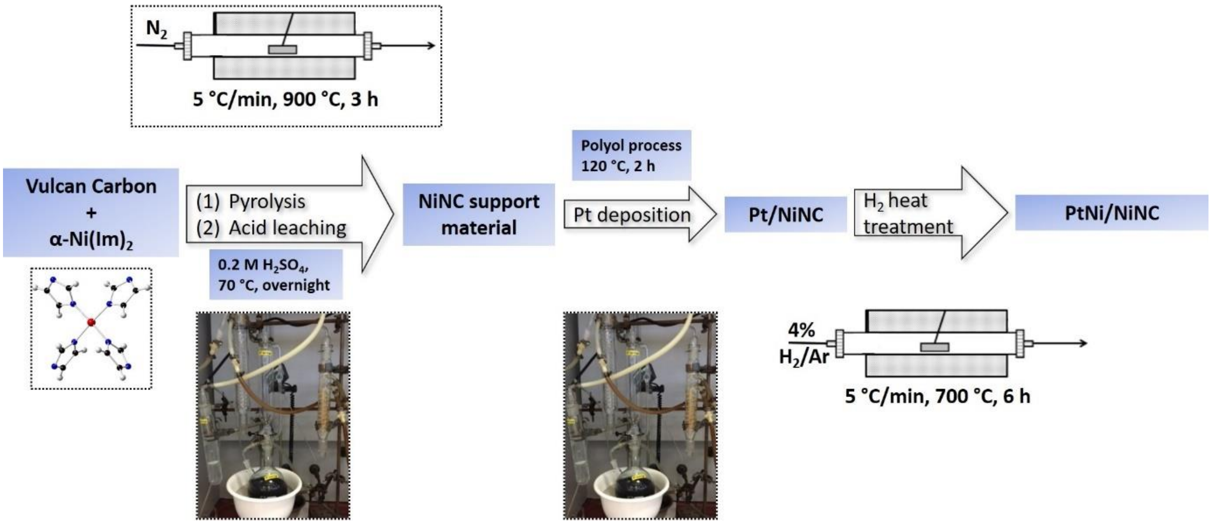

Figure S1. Complete synthetic route toward PtNi/NiNC catalyst material.

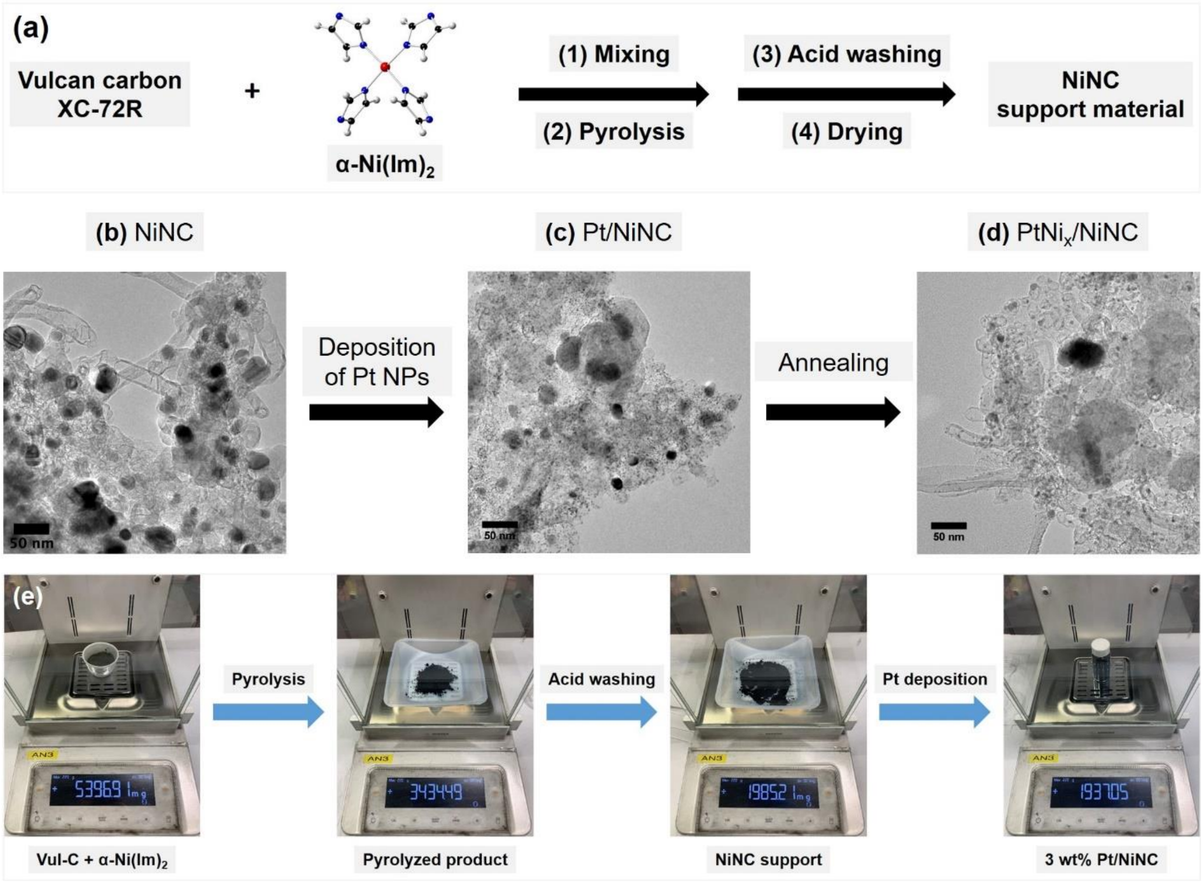

Figure S2. Schematic illustration of the pyrolysis–leaching–deposition–annealing (PLDA) strategy reported in this study and demonstration of its ability to generate Pt/NiNC catalyst at gram-scale in one single batch synthesis. (**a**) Synthetic route and (**b**) TEM image of NiNC support material. (**c**) In situ

growth of 3 wt% Pt NPs onto NiNC support, showing coexistence of smaller Pt NPs and larger Ni NPs encapsulated by thick carbon shells. (**d**) Formation of PtNi/NiNC after annealing. (**e**) Images of the products and their weights at each intermediate step: the starting homogeneous mixture of α-$Ni(Im)_2$ precursor and Vulcan-C, after pyrolysis, acid washing and in situ Pt deposition via polyol process.

Note S1. Importance of the green and scalable synthetic route towards active cathode catalysts for the development of PEMFC technologies.

Although so many remarkable achievements have been achieved in the development of cathode ORR electrocatalysts, approaching to a commercially viable PEMFC technology that is feasible for mass production still requires some further improvements.[4] From a holistic perspective, energy-consuming, toxic and environmentally unfavorable synthetic procedures for the production of catalysts will largely undermine the advantages and attractiveness of this promising and green technology.[5] Focusing solely on the performance of Pt-based ORR catalysts, but disregarding both green chemistry and scalability of their synthetic routes will not suffice to realize the large-scale production of cathode catalysts, an indispensably key constituent at the core of automotive fuel cell. At a research level, several inconspicuous and usually overlooked shortcomings involved in the small-scale “test-tube” synthesis in lab will not be negligible in the large-scale industrial production, and are not minor issues anymore. These shortcomings often include the use of toxic, expensive and hazardous chemical reagents as metal precursors, capping agents, reducing agents and solvents when preparing Pt-based ORR catalysts. Besides, elaborate synthetic strategies, which are sensitive to various experimentally synthetic parameters, make the large-scale preparation even more difficult, if not impossible.[4c, 4d, 6] Consequently, designing a simple, green, robust and up-scalable synthetic route for the large-scale production of highly efficient Pt-based ORR catalyst is an important prerequisite for the genuinely commercial application of automotive fuel cells. As shown in **Figures S2e and 20**, although restricted by the limited volume of reaction flasks used in our lab synthesis, our PLDA method are still able to achieve the gram-scale synthesis in one single batch. Therefore, in terms of cost and green chemistry, our method shows great suitability for mass production.

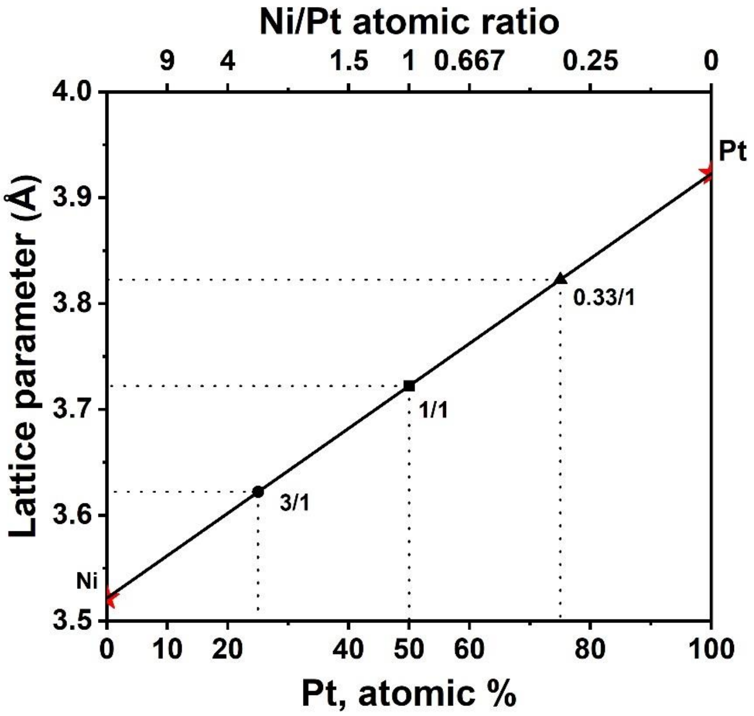

Figure S3. Linear relationship of lattice parameter with alloy composition according to Vegard's rule for solid solutions.[7] Data shown on left and right Y-axis (red star symbols) are lattice parameters of pure Ni and Pt, respectively. Black solid line represents the dependence of lattice parameter on the Pt atomic percentage of Pt-Ni solid solution (disordered) alloys according to Vegard's rule. Solid black circle, square and triangle symbols denote three cardinal compositions of FCC Pt-Ni alloys: $PtNi_3$, Pt_1Ni_1 and Pt_3Ni, respectively. Top X-axis displays the corresponding Ni/Pt atomic ratio at a given Pt atomic percentage derived from bottom X-axis.

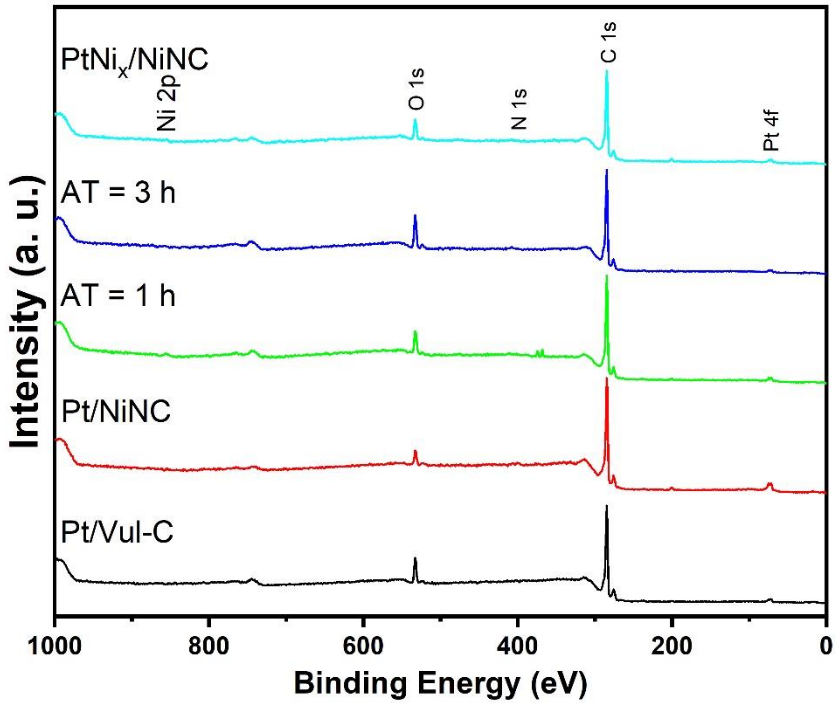

Figure S4. (a) XPS survey spectra of catalyst materials derived from Pt/NiNC with different annealing time, together with one reference sample, Pt/Vul-C. For pristine Pt/NiNC catalyst, annealing time is 0 h. For final PtNi/NiNC catalyst, annealing time is 6 h.

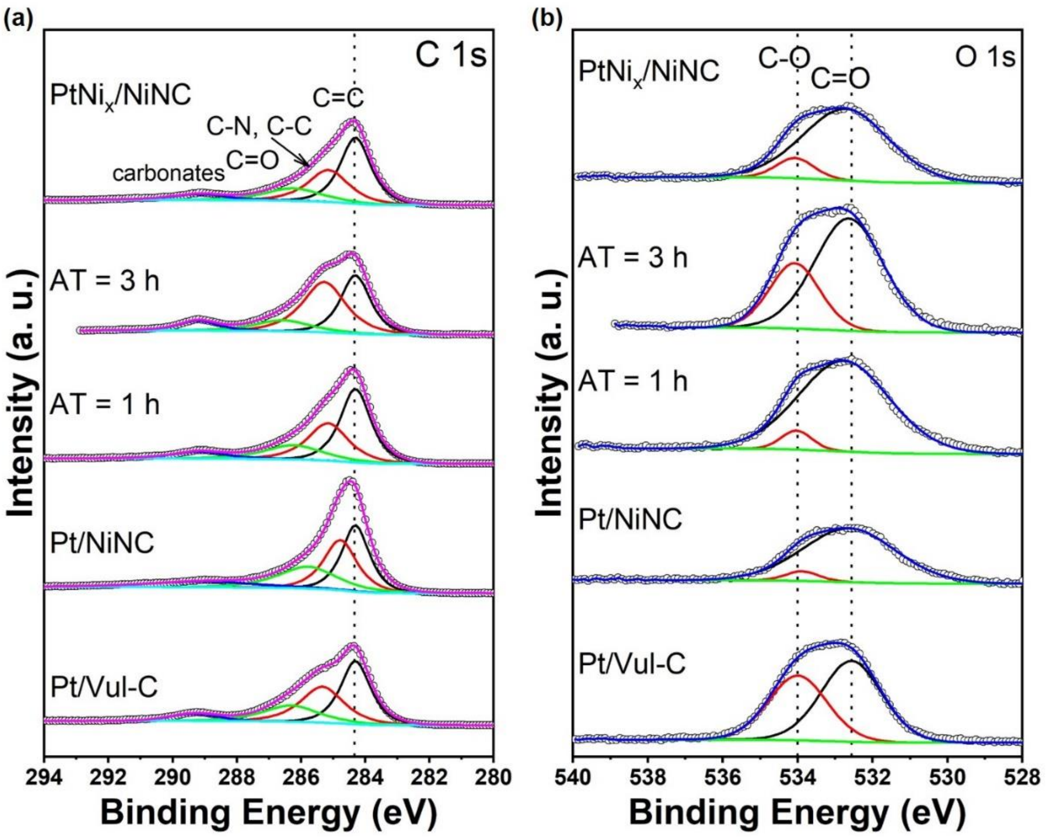

Figure S5. XPS of catalysts, intermediates and reference: (a) C 1s and (b) O 1s spectra of various catalyst materials derived from pristine Pt/NiNC after different annealing time, together with one reference sample, Pt/Vul-C. For pristine Pt/NiNC catalyst, annealing time is 0 h. For final PtNi/NiNC catalyst, annealing time is 6 h. All O 1s spectra consist of two peaks which are assigned to oxygen single bonding (C-O, 534.0 eV) and double bonding (C=O, 532.6 eV) to carbon. All spectra are calibrated with the main peak of C 1s at 284.3 eV. The spectra were fitted using Casa XPS software.

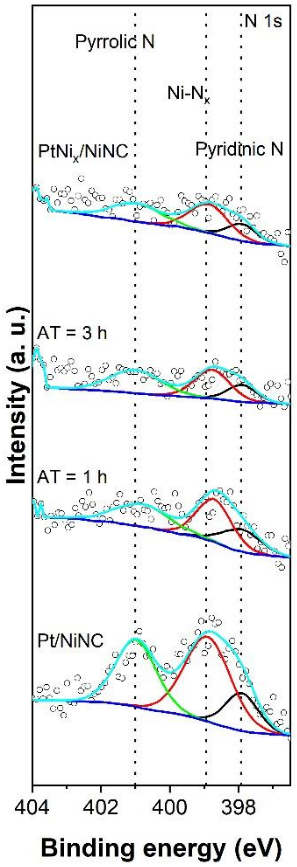

Figure S6. N 1s XPS spectra of catalyst materials derived from pristine Pt/NiNC after different annealing time. For pristine Pt/NiNC catalyst, annealing time is 0 h. For final PtNi/NiNC catalyst, annealing time is 6 h.

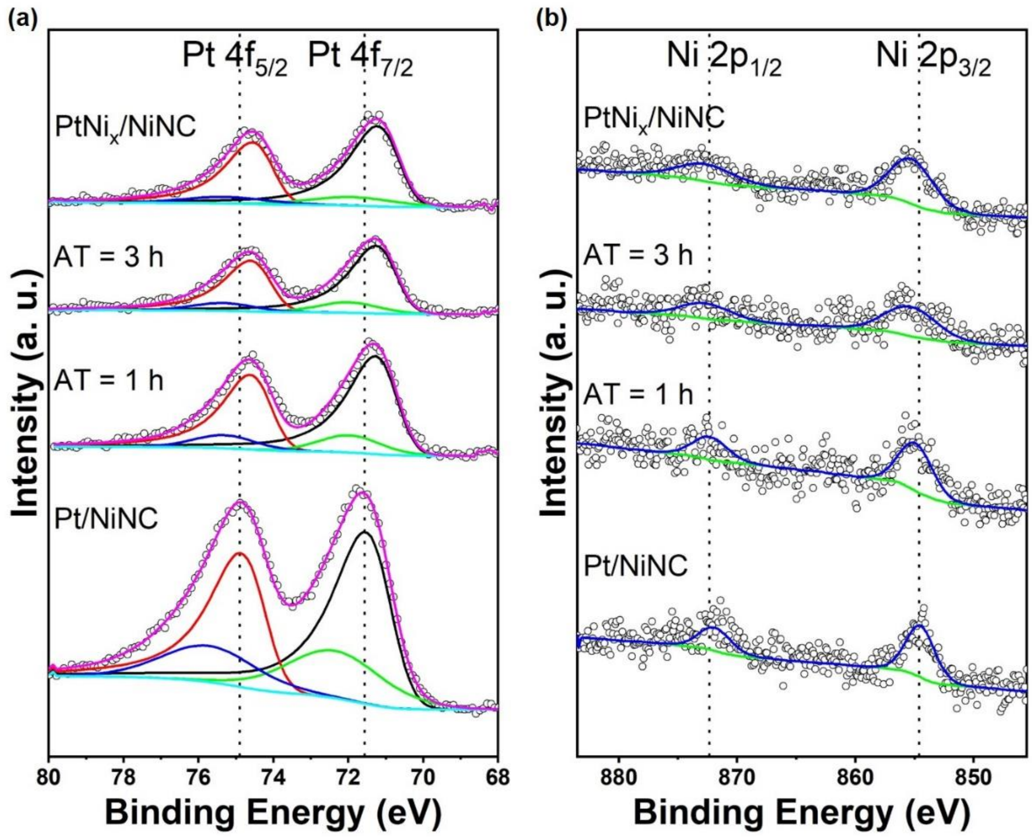

Figure S7. (a) Pt 4f and (b) Ni 2p XPS spectra of catalyst materials derived from Pt/NiNC after different annealing time. For pristine Pt/NiNC catalyst, annealing time is 0 h. For final PtNi/NiNC catalyst, annealing time is 6 h.

Table S1. Elemental compositions (in both at% and wt%) derived from XPS for different catalyst materials. For pristine Pt/NiNC catalyst, annealing time is 0 h. For final PtNi/NiNC catalyst, annealing time is 6 h.

Samples	C (at%)	N (at%)	O (at%)	Pt (at%)	Ni (at%)	C (wt%)	N (wt%)	O (wt%)	Pt (wt%)	Ni (wt%)
Pt/Vul-C	90.44	NA	9.27	0.29	NA	84.17	NA	11.50	4.32	NA
Pt/NiNC	93.14	1.12	5.01	0.61	0.12	83.39	1.17	5.98	8.94	0.53
AT = 1 h	90.26	0.54 ↓	8.79	0.27	0.14	83.83	0.59	10.89	4.07	0.62
AT = 3 h	88.39	0.40 ↓	10.90	0.17	0.13	82.74	0.44	13.60	2.63	0.59
$PtNi_x$/NiNC	91.18	0.44 ↓	7.95	0.25	0.19	85.02	0.47	9.88	3.75	0.87

Table S2. Elemental distribution of different Pt species as derived from the fitting results. For pristine Pt/NiNC catalyst, annealing time is 0 h. For final PtNi/NiNC catalyst, annealing time is 6 h.

Samples	Pt^0 $4f_{7/2}$ position	Pt^0/Pt^{2+}	Ni $2p_{3/2}$ position	Pt^0 (%)	Pt^{2+} (%)
Pt/Vul-C	71.02	2.43	NA	70.88	29.12
Pt/NiNC	71.07	2.66	854.47	72.69	27.31
AT = 1 h	70.88 ↓	5.43 ↑	854.88 ↑	84.45	15.55
AT = 3 h	70.89 ↓	5.98 ↑	855.29 ↑	85.68	14.32
$PtNi_x$/NiNC	70.83 ↓	6.54 ↑	855.21 ↑	86.74	13.26

Table S3. Change of Ni/Pt atomic ratio versus the annealing time in hours. For pristine Pt/NiNC catalyst, annealing time is 0 h. For final PtNi/NiNC catalyst, annealing time is 6 h. By normalizing the total integral areas of Ni 2p and Pt 4f regions to their corresponding relative sensitivity factors (RSFs), the Ni/Pt atomic ratios were obtained from XPS spectra.

Samples	Total Ni 2p integral area	Total Pt 4f integral area	Ni/Pt atomic ratio
Pt/NiNC	2485.8	9524.4	0.20
AT = 1 h	2659.1	3957.8	0.51 ↑
AT = 3 h	2523.6	2553.3	0.74 ↑
$PtNi_x$/NiNC	3226.7	3144.6	0.77 ↑

Table S4. Metal contents in weight percentage determined by ICP for catalyst materials derived from Pt/NiNC, as well as Pt/Vul-C reference material.

Samples	Pt wt% (ICP)	Ni wt% (ICP)
Pt/Vul-C	3.01	NA

Pt/NiNC	3.14	6.91
AT = 1 h	2.96	6.57
AT = 3 h	3.32	7.56
$PtNi_x$/NiNC	3.23 ↑	7.42 ↑

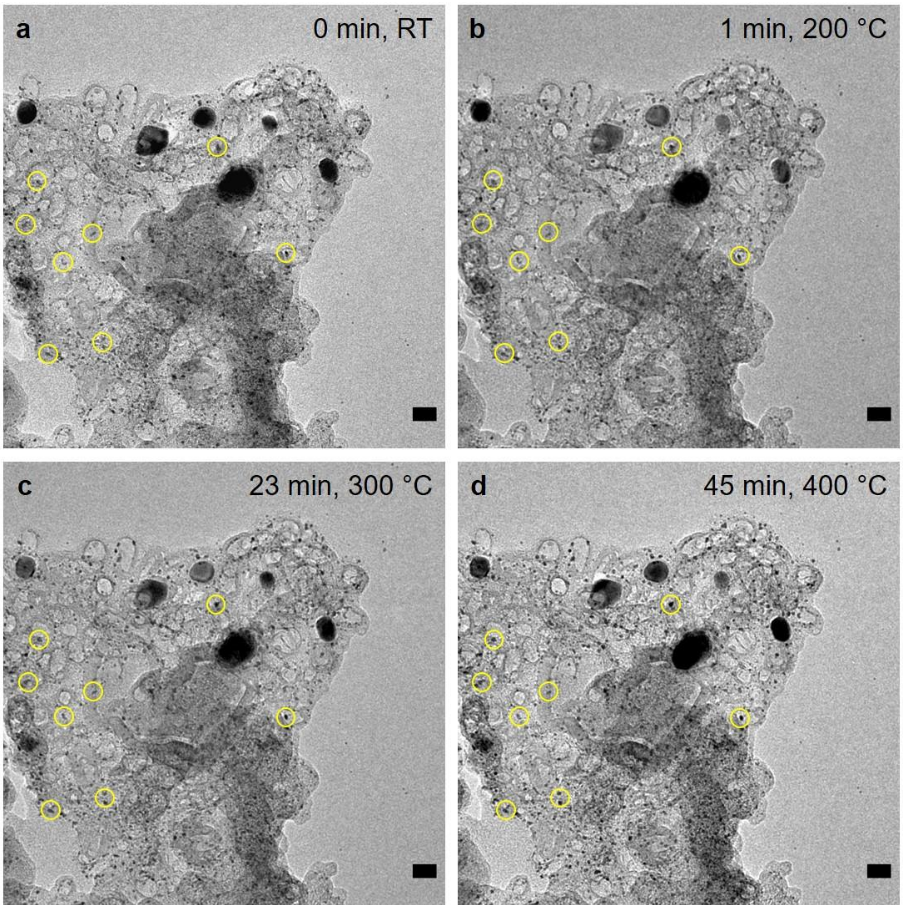

Figure S8. *In situ* TEM image series taken below 500 °C. At lower temperatures, small Pt NPs as marked by yellow circles either agglomerated slightly or become rounded in shape, whereas Ni NPs are stable. Scale bar: 20 nm.

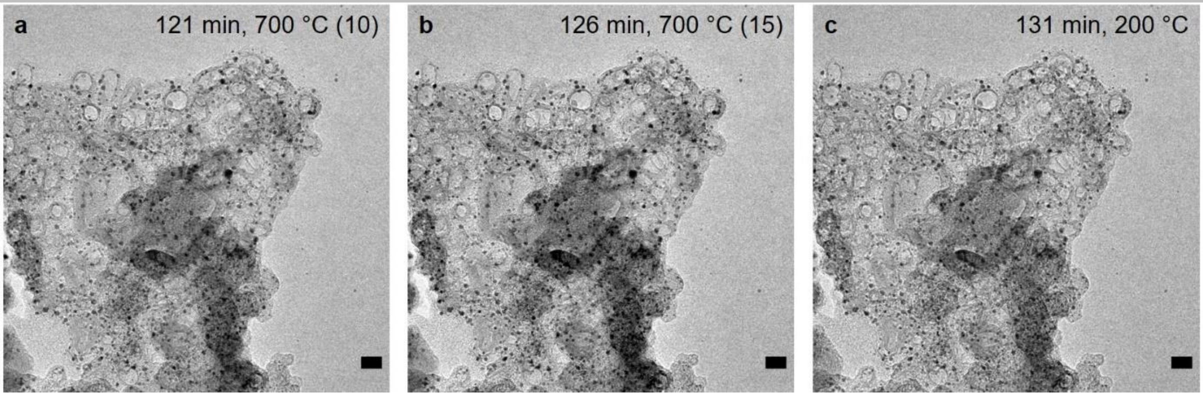

Figure S9. Tracking morphological changes in real time. *In situ* TEM images recorded during a temperature hold of 15 min at 700 °C (a, b) and after subsequent cooling to 200 °C (c). The numbers in parentheses indicate the holding time in minutes at 700 °C. Scale bar: 20 nm.

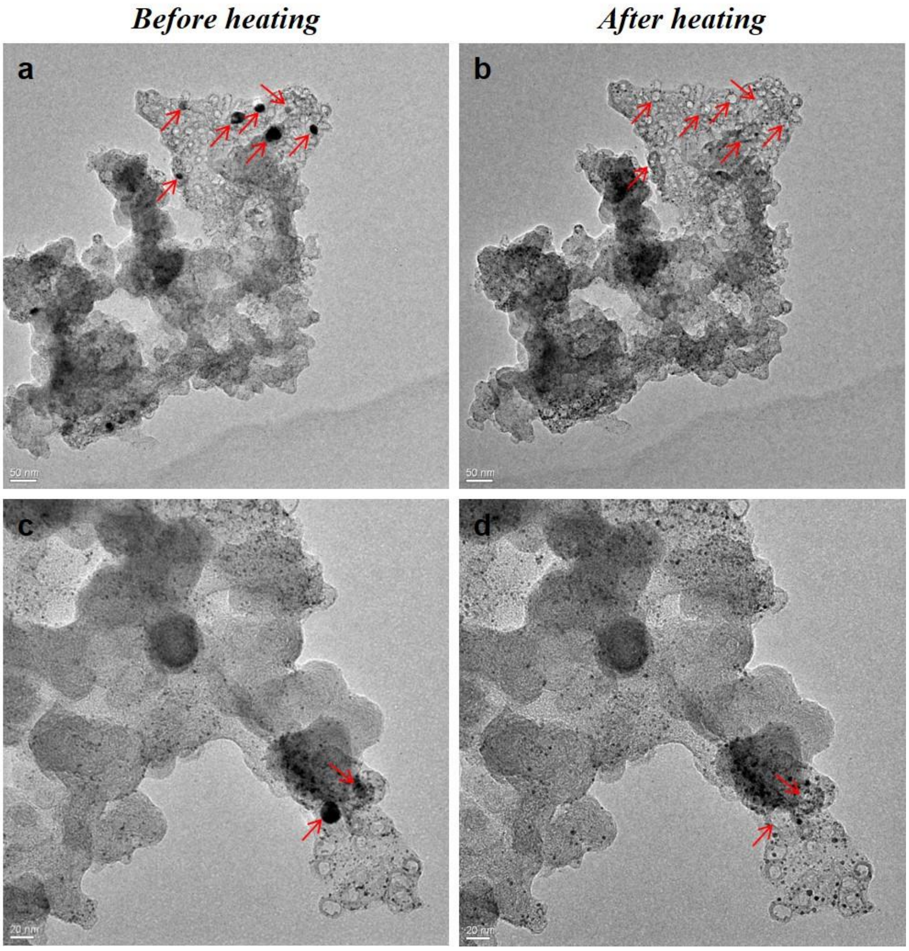

Figure S10. Exclusion of beam damage misinterpretations. Impact of electron beam during *in situ* heating. TEM images taken (a, c) before and (b, d) after the same heating procedure from other two regions, which neither were exposed to electron beam during *in situ* heating experiment. Herein,

similar changes are observed as in main text: after heating, these Ni NPs marked by red arrows disappear completely. From this beam control experiment, we could conclude that all changes observed in our *in situ* heating experiment result from heating, rather than electron beam damage.

Note S2. Experimental verification of absence of beam damage effect (relating to **Figure S10** for Pt/NiNC and **Figure S18** for Pt/NiNC-1)

In order to check for the influence of electron beam damage, we recorded TEM images of two other catalyst regions only briefly before and after completion of our *in situ* heating experiment (**Figure S10** for Pt/NiNC and **Figure S18** for Pt/NiNC-1). As very similar morphological changes to Pt and Ni NPs were observed on the non-irradiated regions, we are confident that the diffusion and shrinkage of Ni atoms and subsequent alloy formation originate from the thermal treatment rather than from beam effects.

Note S3. Thermal annealing of Pt/NiNC-1 in absence of C-encapsulated Ni NPs (relating to **Figures S11–19**)

To verify the dominant role of metallic Ni NPs as the source of mobile Ni atoms alloying with Pt particles during the thermal formation of PtNi nanoalloy NPs, we prepared a reference material in analogy to Pt/NiNC (synthetic process see **Figures S11, 12**), denoted as "Pt/NiNC-1", which had a lower Ni content of only 1 wt% and contained no metallic Ni NPs present (**Figure S11b**). Key difference compared to the synthesis of Figure 1 was the preparation and use of the lower Ni-wt% support, NiNC-1. Pt deposition on NiNC-1 and further thermal annealing of Pt/NiNC-1 was identical.

After thermal annealing of the Pt/NiNC-1 catalyst, the right shift of the Pt(111) reflection in **Figure S13** suggested the formation of a bimetallic PtM alloy phase with a second metallic component "M". But due to the fact that both Zn (3.6 wt%) and Ni (1.2 wt%) coexist in the pyrolyzed "NiNC-1" support material, it is unclear which metal element took part in the alloy formation and at what proportions if both do. This is the reason why we denote the annealed catalyst as "PtM_x/NiNC-1", in which M represents either Zn or Ni. Importantly, the voltammetric CO stripping and associated CV measurements in alkaline electrolyte (**Figure S19a and b**) unambiguously indicate the absence of "Ni"-type CO stripping peaks

and Ni^{2+}/Ni^{3+} redox peaks. This implies that Ni cannot be involved in the formation of the PtM_x alloy phase. Overall, this control experiment confirms that Ni NPs act as the main source of Ni species in the process of alloy formation.

Table S5. Metal contents in wt% determined by ICP for catalyst materials derived from NiNC-1 support.

Samples	Pt wt% (ICP)	Ni wt% (ICP)	Zn wt% (ICP)
NiNC-1	NA	1.20	3.60
Pt/NiNC-1	2.76	1.09	2.70
PtM_x/NiNC-1	3.17 ↑	1.26 ↑	2.70

Owing to a mass loss caused by unstable volatile species during the annealing process, the contents of Pt and Ni in annealed samples are slightly increased.

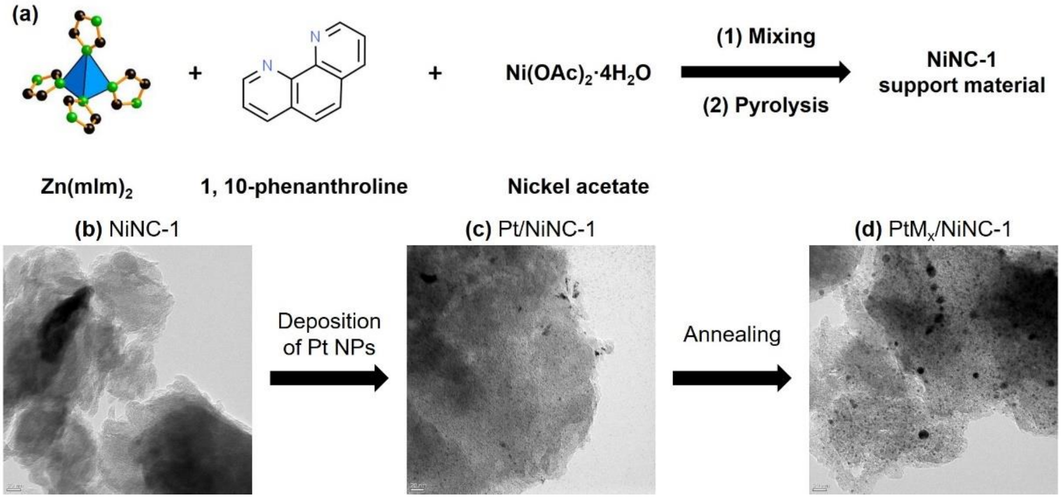

Figure S11. Synthetic scheme and TEM images of the NiNC-1 support-based materials at three different steps. (**a**) Synthetic route of the blank NiNC-1 support material. (**b**) Initial NiNC-1 support material, showing no presence of Ni NPs. (**c**) In situ growth of 3 wt% Pt NPs on NiNC-1 support through polyol process. (**d**) Formation of PtM_x/NiNC-1 after annealing treatment. Scale bar: 20 nm.

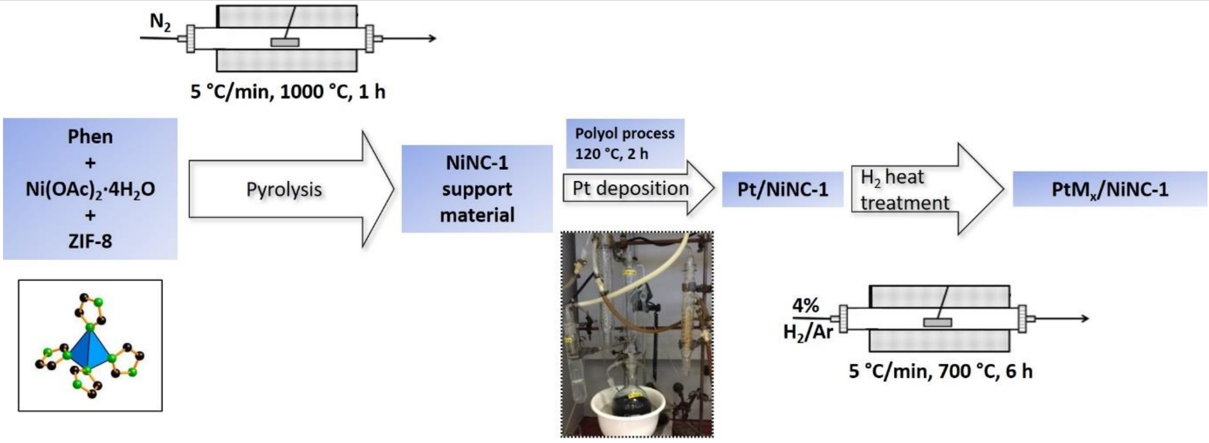

Figure S12. Complete synthetic route toward PtM_x/NiNC-1 catalyst material.

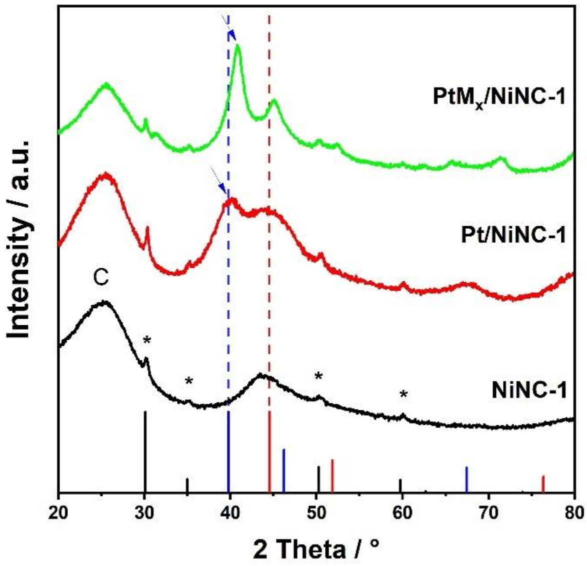

Figure S13. XRD patterns of the as-prepared Ni NP-free NiNC-1 support, pristine Pt/NiNC-1 material obtained after deposition of Pt NPs, and the final annealed PtM_x/ NiNC-1 samples. Blue arrows clearly show the successful growth of Pt NPs on NiNC-1 support and the evolution from pure Pt NPs to PtM_x alloy phase.

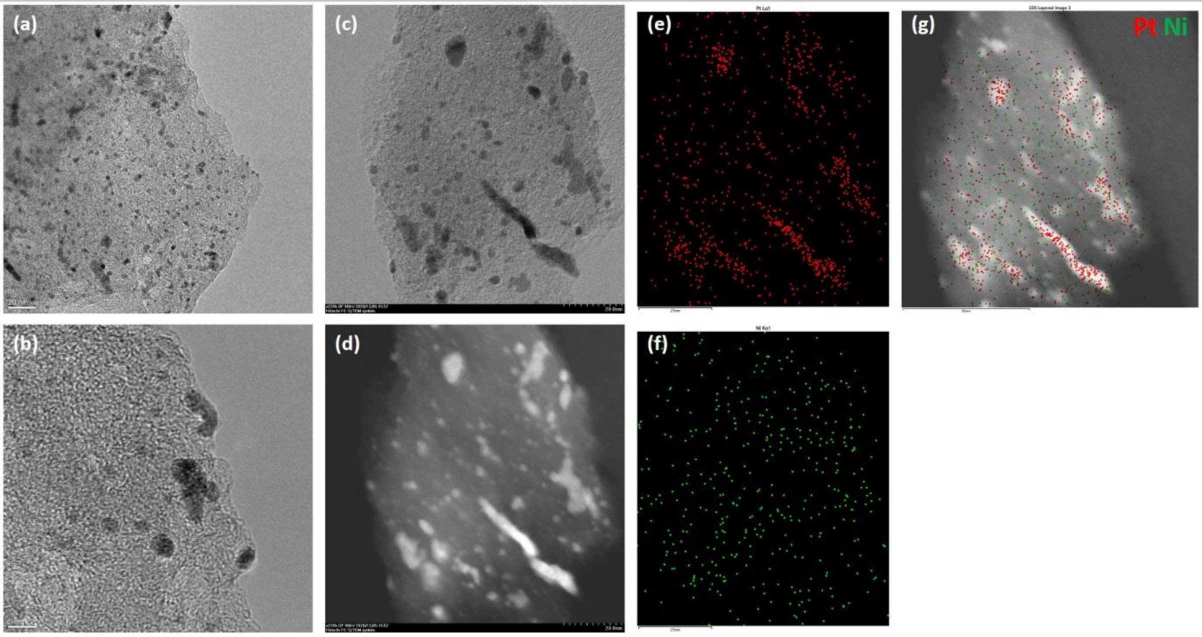

Figure S14. HRTEM images of pristine Pt/NiNC-1 catalyst at lower (a) and higher (b) magnifications. HAADF STEM images at both (c) bright field (BF) and (d) dark field (DF). STEM-EDX compositional mapping profiles of the same area as in (c and d): (e) single Pt, (f) single Ni, and (g) overlapping of Pt (red) and Ni (green).

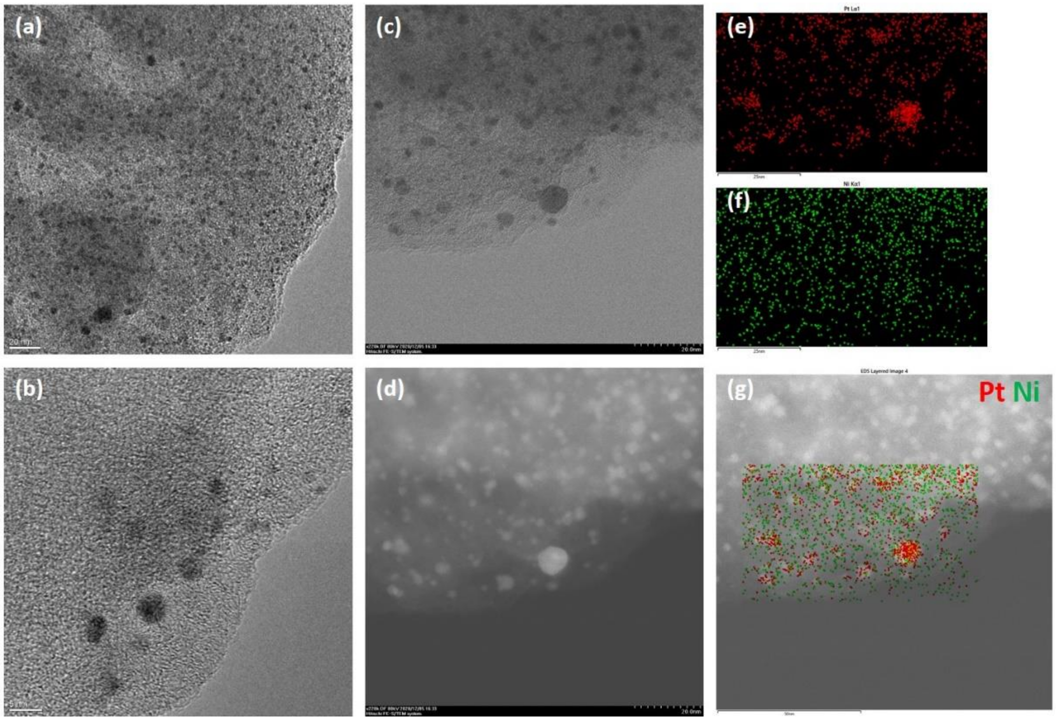

Figure S15. HRTEM images of annealed PtM_x/NiNC-1 catalyst at lower (a) and higher (b) magnifications. HAADF STEM images at both (c) bright field (BF) and (d) dark field (DF). STEM-EDX

compositional mapping profiles of the same area as in (c and d): (e) single Pt, (f) single Ni, and (g) overlapping of Pt (red) and Ni (green).

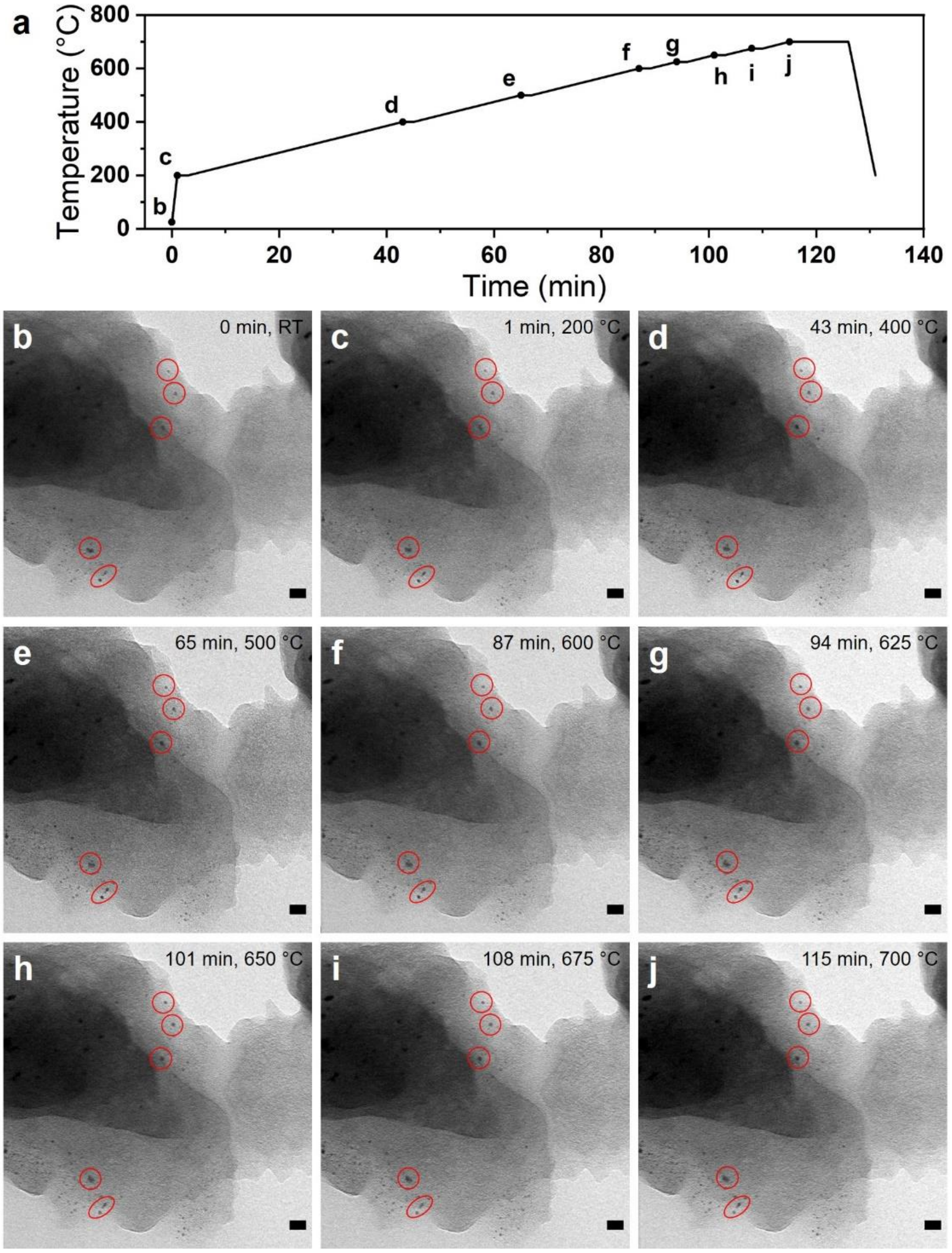

Figure S16. *In situ* TEM imaging of the catalyst during heating from 25 °C (RT) to 700 °C under vacuum condition. (a) Temperature profile over time with marked points corresponding to the images in (b–g). Starting from 200 °C, temperature was increased in step of 100 °C (5 K/min). The specimen was annealed for 2 min at each step below 600 °C. Between 600 and 700 °C, temperature was increased in step of 25 °C and kept constant at each step for 2 min. And sample temperature was kept at final

700 °C for 11 min. Red circles in b–j mark the Pt NPs slightly agglomerated during thermal annealing. Scale bar: 20 nm.

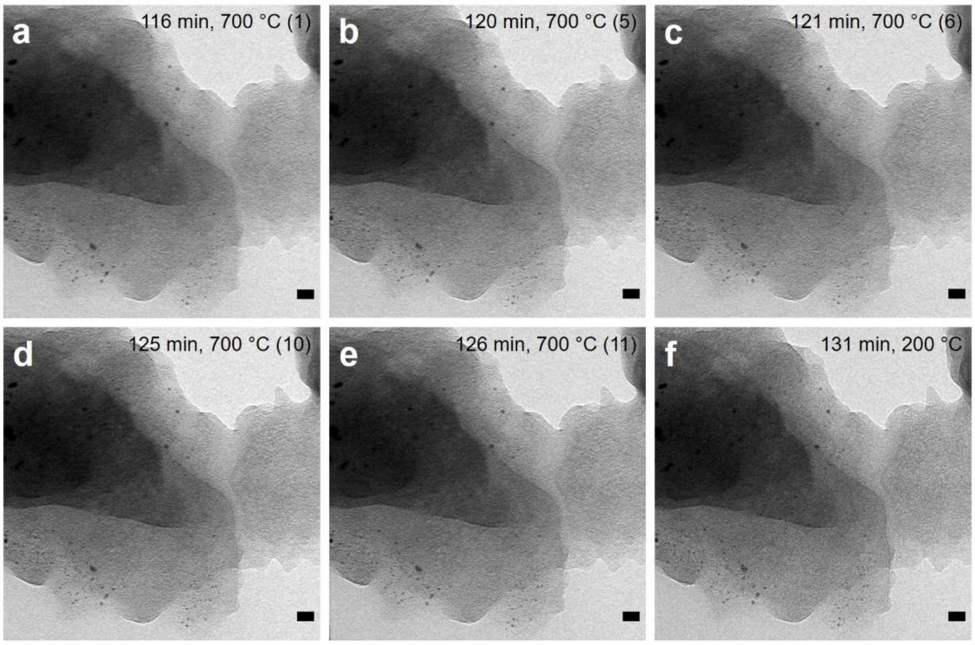

Figure S17. *In situ* TEM images recorded during a temperature hold of 11 min at 700 °C (a–e) and after subsequent cooling to 200 °C (f). The numbers in parentheses indicate the holding time in minutes at 700 °C. Scale bar: 20 nm.

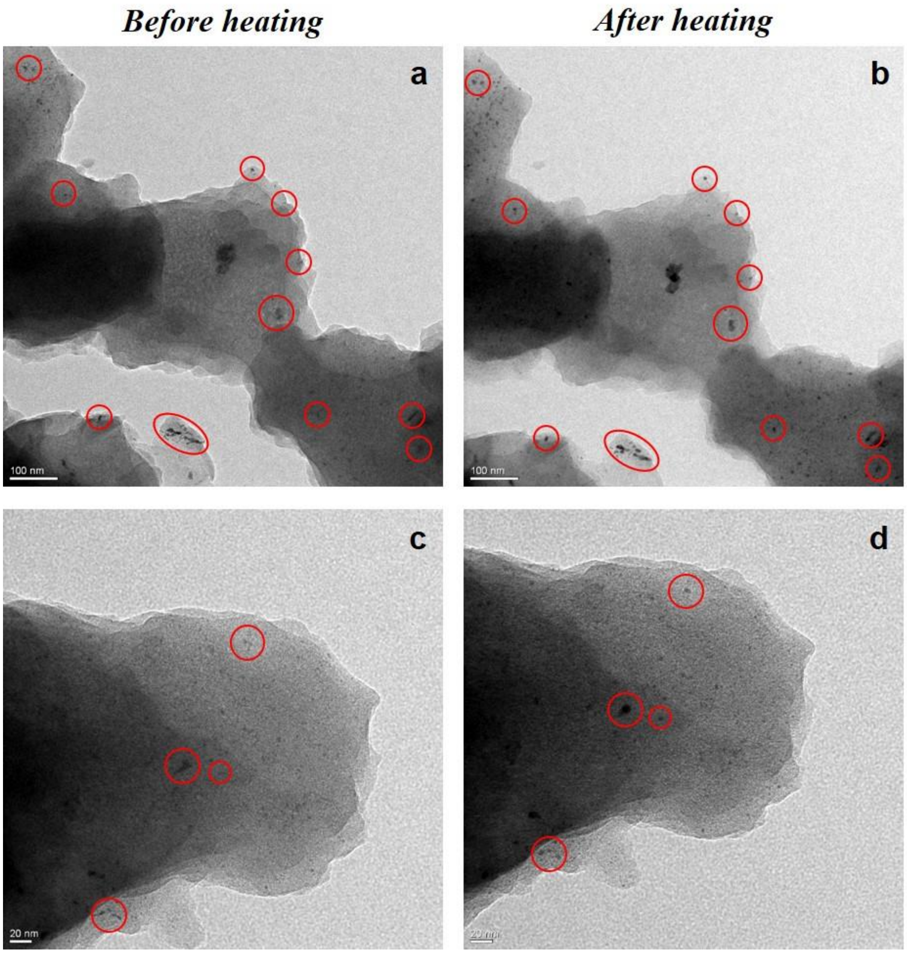

Figure S18. Impact of electron beam during in situ heating and exclusion of beam damage misinterpretations. TEM images taken (a, c) before and (b, d) after the same heating procedure from other two regions, which neither were exposed to electron beam during *in situ* heating experiment. Herein, similar changes are observed as in **Figure S16**. From this beam control experiment, we could conclude that all changes observed in our *in situ* heating experiment result from heating, rather than electron beam damage.

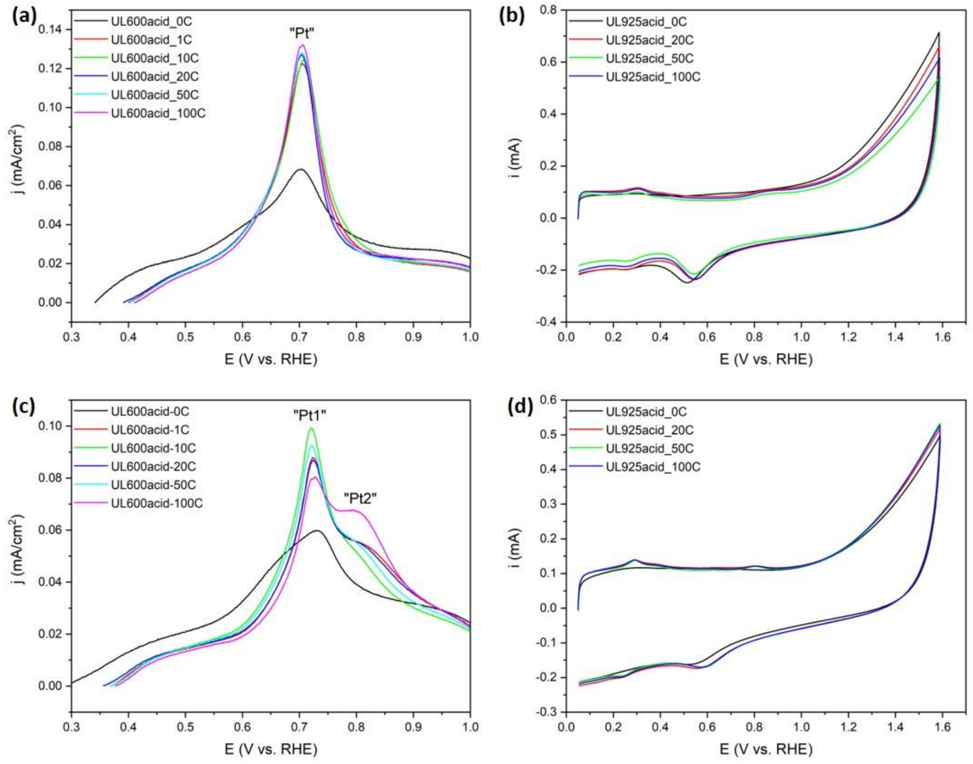

Figure S19. (a, c) Pt surface area-normalized and background-subtracted CO stripping voltammetry and (b, d) CV curves in 0.1 M KOH solution for a series of catalyst films undergoing pre-treatment in acidic solution with different potential cycling numbers: (a, b) annealed PtM_x/NiNC-1 and (c, d) pristine Pt/NiNC-1 samples, respectively.

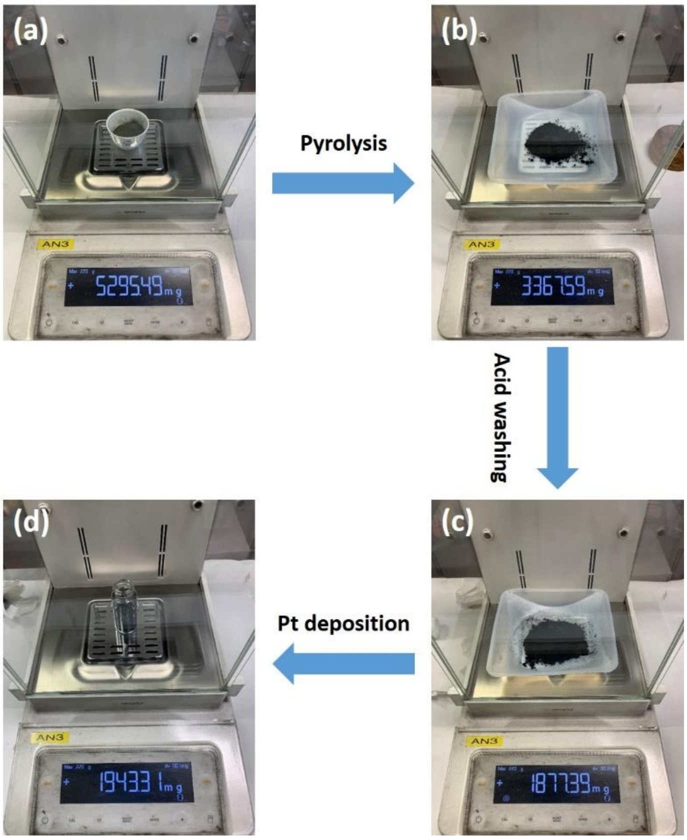

Figure S20. Images of the product and its weight at each intermediate step: (a) the starting material, homogeneous mixture of α-Ni(Im)$_2$ precursor and Vulcan carbon in a weight ratio of 10:1; (b) after pyrolysis; (c) after acid washing and (d) after 5 wt% Pt deposition via polyol process.

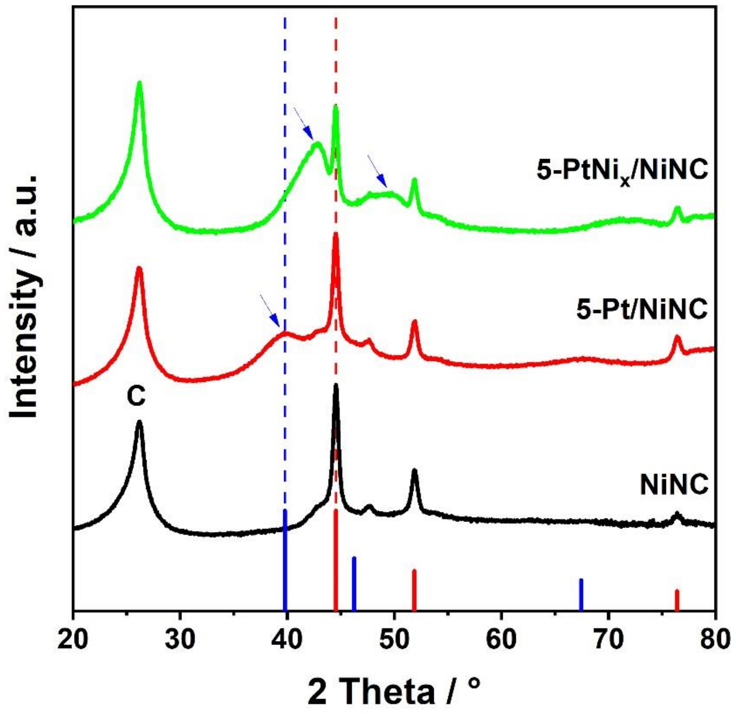

Figure S21. XRD pattern of blank NiNC support, pristine 5-Pt/NiNC and annealed 5-PtNi/NiNC catalyst materials.

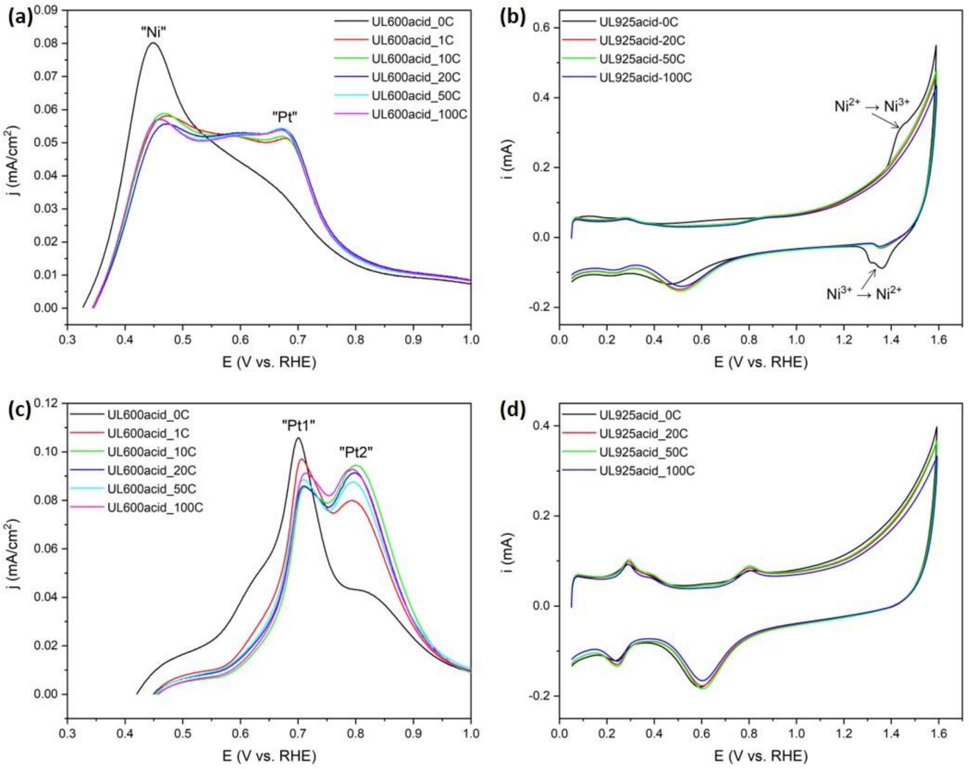

Figure S22. (a, c) Pt surface area-normalized and background-subtracted CO stripping voltammetry and (b, d) CV curves in 0.1 M KOH solution for a series of catalyst films undergoing pre-treatment in acidic solution with different potential cycling numbers: (a, b) annealed 5-PtNi$_x$/NiNC and (c, d) pristine 5-Pt/NiNC samples, respectively.

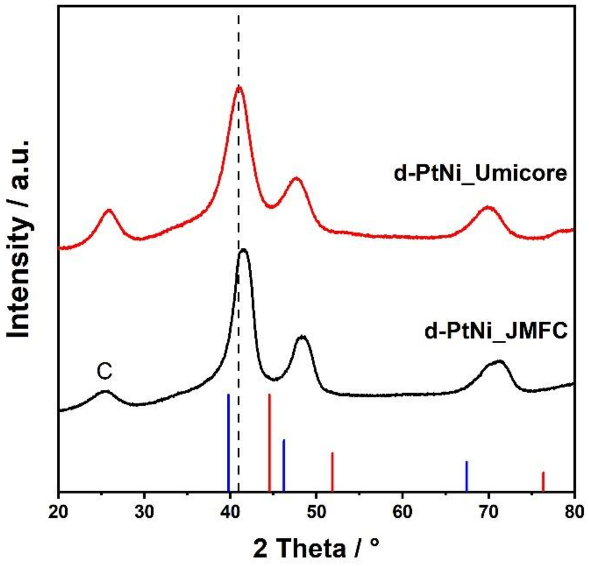

Figure S23. XRD patterns of two d-PtNi reference samples from JMFC and Umicore, respectively. For d-PtNi_JMFC sample, Pt content is 28.6 wt%, and Ni content is 4.7 wt%, with a Pt/Ni atomic ratio of $Pt_{1.83}Ni$. For d-PtNi_Umicore sample, Pt content is 28.13 wt%, and Ni content is 3.02 wt%, with a Pt/Ni atomic ratio of $Pt_{2.80}Ni$. Due to its higher Ni content in d-PtNi_JMFC, according to Vegard's rule (**Figure S3**), it is expected that Pt(111) diffraction peak of d-PtNi_JMFC would shift to higher 2theta degree. Then this is further confirmed by our XRD results. The vertical dashed line is present as a guide for eyes.

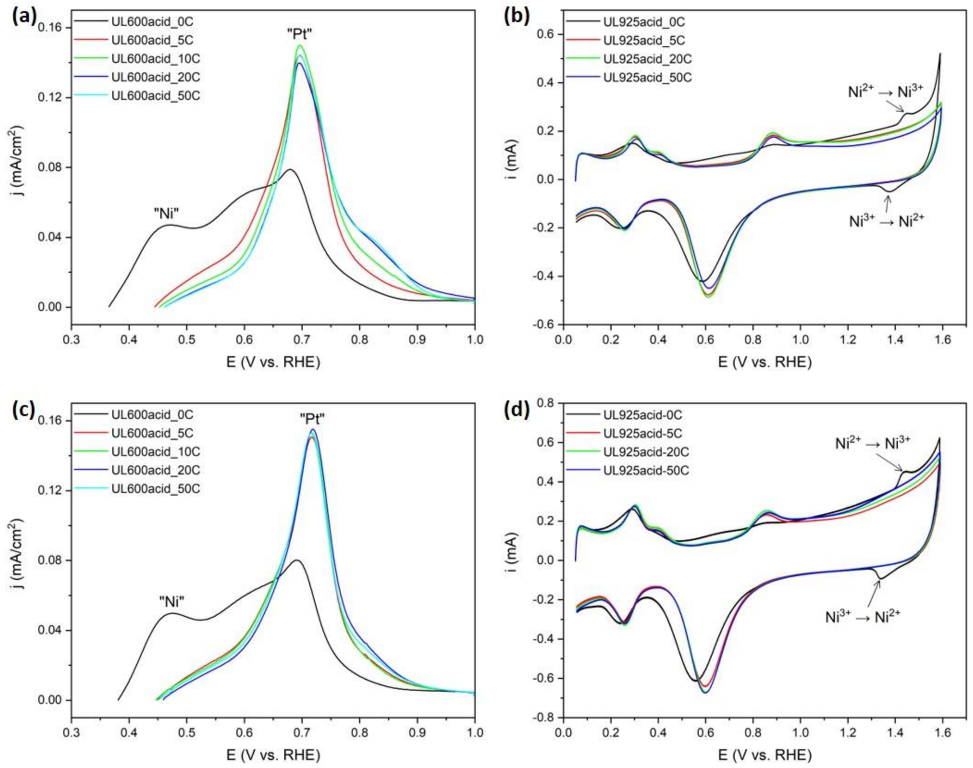

Figure S24. (a, c) Pt surface area-normalized and background-subtracted CO stripping voltammetry and (b, d) CV curves in 0.1 M KOH solution for a series of catalyst films undergoing pre-treatment in acidic solution with different potential cycling numbers: (a, b) d-PtNi_JMFC and (c, d) d-PtNi_Umicore samples, respectively.

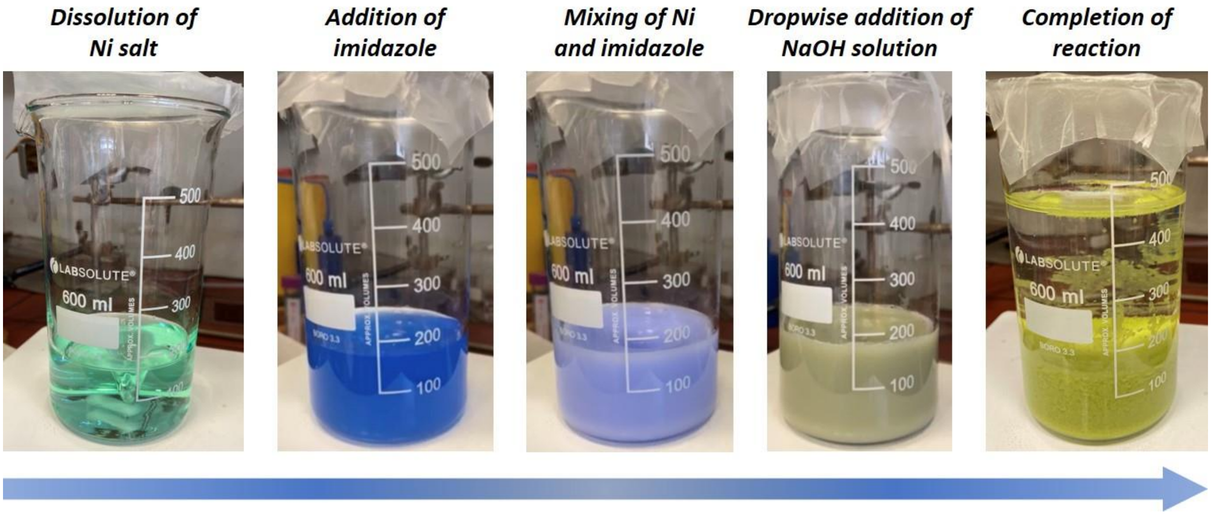

Figure S25. Colour change of the reaction system during reaction time. Room temperature synthesis of α-$Ni(Im)_2$ precursor.

Note S4. Quantitative estimation of the contribution of accessible NiN_x sites to the overall activity of Pt/NiNC catalyst.

First of all, from a qualitative standpoint, due to the much lower intrinsic ORR activity of NiN_x sites than Pt, it is expected that these ORR-inactive NiNC sites barely contribute to the overall activity of the Pt/NiNC catalyst. Previous DFT studies suggest that this poor ORR activity results from the weak O* adsorption energy on Ni site.[8] Then such a hypothesis is corroborated by our quantitative estimation result as shown below.

The geometric catalyst loading of Pt/NiNC on the GC electrode is

$$L_{catal} = \frac{\frac{5.83\ mg}{4.99\ mL} \times 10\ \mu L}{0.19625\ cm^2} = 59.5\ \mu g/cm^2$$

And the geometric mass loading of accessible NiN_x sites is

$$L_{Ni\ site} = L_{catal} \times \omega_{Ni\ site} = 59.5 \times 0.53\% = 0.32\ \mu g/cm^2$$

Considering that most of Ni in NiNC support is Ni NPs, which are completely encapsulated by thick carbon layer, we prefer to use the Ni content derived from XPS technique, rather than that derived from ICP technique, as accessible Ni site, $\omega_{Ni\ site}$. We would like to point to the difference in the distinct probing characteristics of both techniques: XPS is surface-sensitive with detection depth at specific photo energy, but ICP is a bulk method probing the total content of Ni species both from surface and buried under the thick carbon layer.

And the geometric molar amount of accessible NiN_x site is

$$n_{Ni\ site} = \frac{L_{Ni\ site}}{M_{Ni}} = \frac{0.32\ \mu g/cm^2}{58.69\ \mu g/\mu mol} = 5.5 \times 10^{-3}\ \mu mol/cm^2 = 5.5 \times 10^{-9}\ mol/cm^2$$

Where M_{Ni} is the molar mass of nickel.

Thus, the geometric kinetic current density from accessible NiN_x sites is

$$j_{Ni\,site} = n_{Ni\,site} \times N_A \times TOF_{Ni\,site}[e\,site^{-1}\,s^{-1}] \times e$$
$$= 5.5 \times 10^{-9} \times 6.02 \times 10^{23} \times 1.6 \times 10^{-19} \times TOF\,[A/cm^2]$$
$$= 53 \times 10^{-5} \times TOF\,[A/cm^2] = 0.53 \times TOF\,[mA/cm^2]$$

Where TOF is the turnover frequency, N_A is the Avogadro's constant, e is the electric charge of one electron.

The overall kinetic current density, j_K, was calculated according to Koutecky-Levich equation:

$$\frac{1}{j_K} = \frac{1}{j_{0.8}} - \frac{1}{j_D} = \frac{1.74 \times 4.82}{4.82 - 1.74} = 2.74\,mA/cm^2$$

Where $j_{0.8}$ is the current density at 0.8 V_{RHE}, and j_D is the diffusion current density.

Finally, the percentage contribution of accessible NiN_x sites to the overall activity can be calculated as:

$$= 100\% \times \frac{j_{Ni\,site}}{j_K} = 100\% \times \frac{0.53 \times TOF}{2.74} = 19.3 \times TOF\ (\%)$$

The above calculations indicate that the ORR activity contribution from NiN_x sites has a linear relationship with the intrinsic TOF value of NiN_x site. Due to the fact that the NiNC catalyst does not demonstrate any CO chemisorption, quantification of the mass-based site density derived from CO pulse chemisorption is not possible to perform. Consequently, one could not obtain the TOF value of NiN_x sites using CO sorption method. Although at present there is no TOF value of NiN_x sites from literatures, for comparison, next we use the TOF values of its iron, cobalt and copper counterparts from literature to estimate their respective percentage contribution to the overall ORR activity.[8b] This could provide us with some indications about the actual contribution of NiN_x sites. Assuming that these accessible NiN_x sites are substituted by same amount of their Fe counterparts, despite its highest intrinsic TOF value, it only contributes to around 3% of the overall activity. Considering the much lower intrinsic activity of NiN_x site than FeN_x site,[8] we conclude that NiN_x sites barely contribute to the overall activity.

Table S6. Assumed TOF values at 0.8 V_{RHE} in 0.1 M $HClO_4$ electrolyte and ensuing $j_{Ni\,site}$ and its contribution to the overall activity.

TOF (e site^{-1} s^{-1}) [a]	$j_{Ni\,site}$ (mA/cm^2)	Percentage contribution to overall j (%)

0.15 (Fe)	0.08	2.91
0.029 (Co)	0.02	0.56
0.006 (Cu)	0.00	0.12

[a] RDE measurement conditions: temperature of 25 °C; geometric catalyst loading of 800 μg/cm^2; scan rate of 5 mV/s; rotation of 1600 rpm; negative going scan was used to evaluate the ORR activity.

Note S5. Advantage of our NiNC support versus conventional Vulcan carbon.

Owing to the mesoporous structure and Ni- and N-doped feature, our NiNC support exhibit several advantages versus conventional Vulcan carbon support. First, compared with undoped Vulcan carbon, NiNC support bears nitrogen functional groups that interact with the ionomer to promote its homogeneous distribution. Owing to the Coulombic attraction between the ionomer and N groups on the NiNC support, NiNC support could ensure very uniform coverage of the ionomer.[2a] Second, carbon supports with a mesoporous structure (with a pore opening of 4–7 nm) promise excellent ORR activity and transport properties simultaneously.[9] Third, the highly graphitized carbon is more resistant to carbon corrosion.

For Pt/NiNC catalyst, due to the difficulty in obtaining an uniform catalyst film without the addition of Nafion ionomer, we only measured its performance with the addition of Nafion ionomer. As shown in Figure 5a and d, for Pt/NiNC, albeit with ionomer, it still exhibits measurable CO signal and H_{upd} features, indicating its advantages over conventional Vulcan carbon.

To evaluate the effect of ionomer distribution on the electrochemical performance of Pt/Vul-C catalyst, we measure its ORR performance with and without the addition of Nafion ionomer. When ionomer is absent, Pt surface is free of ionomer and the poisoning effect is absent. As shown in **Figure S26**, upon the addition of Nafion ionomer, both CO signal and H_{upd} features of Pt/Vul-C were inhibited due to the poisoning effect on Pt surface. Carbon monoxide strongly adsorbs on metallic Pt surface. The weakening of CO signal demonstrated that a faction of Pt surface was fully covered by the thick ionomer layer and was not electrochemically-accessible anymore.

As shown in **Figure S26b**, both kinetic current at 0.9 V_{RHE} and diffusion current became smaller when ionomer was added. In the diffusion limiting regime, Pt particles hidden under the thick ionomer layer exhibit restricted protonic and reactant gas access, thus showing lower diffusion current density at low potential. In the kinetic regime, sulfonate groups in the perfluorosulfonic acid (PFSA) ionomer could also poison Pt surface, thus lowering its intrinsic activity.

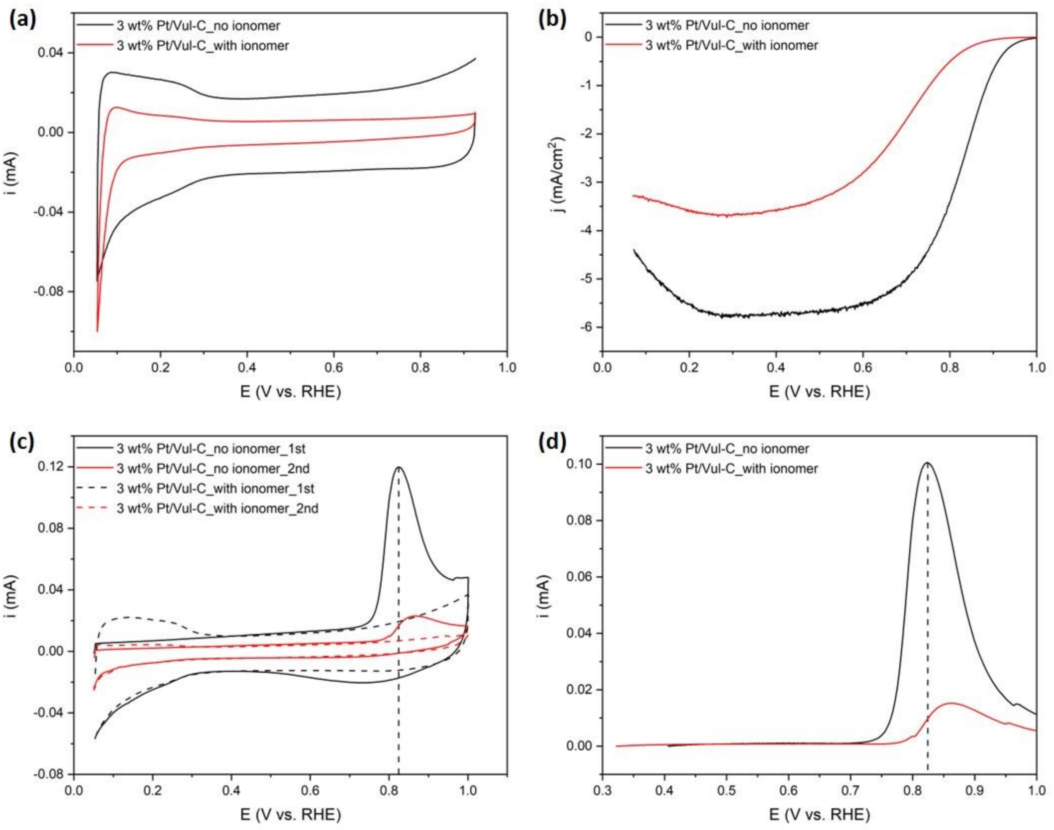

Figure S26. RDE measurements of Pt/Vul-C reference catalyst both with and without the addition of Nafion ionomer: (a) CV, (b) ORR polarization, (c) first and second scans of CO striping curves and (d) background-subtracted CO stripping voltammetry curves.

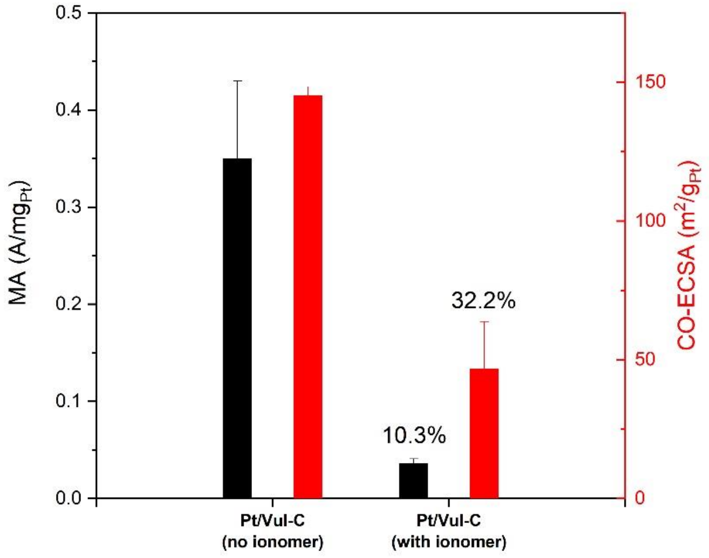

Figure S27. Comparison of mass activities and CO-ECSA of Pt/Vul-C with and without the addition of Nafion ionomer.

Table S7. Comparison of mass activities, specific activities and CO-ECSA of Pt/Vul-C with and without the addition of Nafion ionomer.

Samples	MA (A/mg$_{Pt}$)	SA (mA/cm^2)	CO-ECSA (m^2/g$_{Pt}$)
Pt/Vul-C (no ionomer)	0.35 ± 0.08	0.24 ± 0.05	145.2 ± 3.2
Pt/Vul-C (with ionomer)	0.036 ± 0.005 (10.3%)	0.083 ± 0.027 (34.6%)	46.7 ± 17.0 (32.2%)

Note S6. *In situ* TEM vs *ex situ* tube furnace thermal annealing

From XRD pattern as shown in Figure 2a and **Figure S28**, after *ex situ* thermal annealing under ambient pressure for 6 h, the PtNi/NiNC catalyst after chemical dealloying continued to show weak diffraction peaks corresponding to crystalline Ni NPs. With an even shorter heating protocol, the *in situ* thermal annealing in the TEM holder appears to induce more severe changes on our catalyst material compared with *ex situ* annealing. Therefore, we conclude that *in situ* heating condition is much harsher

than *ex situ* one, possibly due to the ultralow pressure under vacuum condition. Although there still exists an unavoidable gap between *in situ* heating and *ex situ* annealing experiments, these *in situ* TEM experiment could allow us to obtain some valuable information about how our catalyst material evolves during thermal annealing. In terms of this discrepancy, *in situ* environmental TEM (ETEM) technique could provide a gaseous environment and thus come more closer toward realistic *ex situ* annealing conditions.[10]

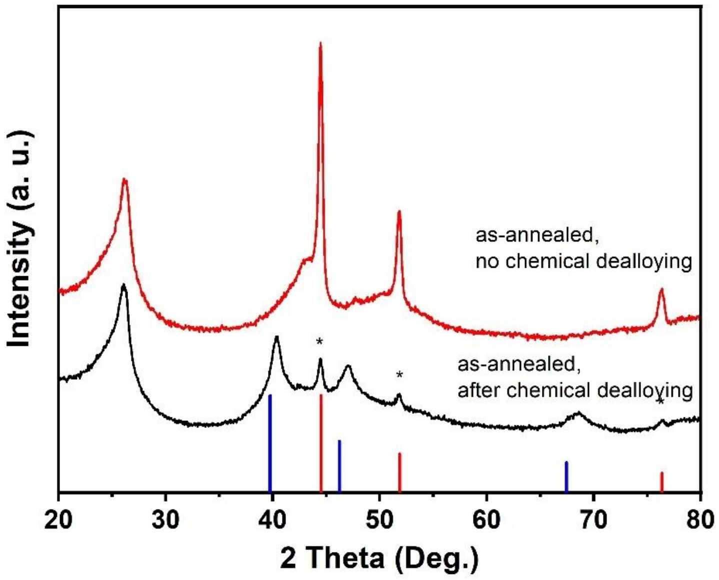

Figure S28. In chemical dealloying, annealed PtNi/NiNC catalyst was treated with 0.1 M $HClO_4$ solution at 80 °C for 4 h and washed with deionized water for several times. After freeze-drying overnight, the material was annealed under 4% H_2/Ar at 500 °C for 1 h. After chemical dealloying treatment, Ni diffraction peaks as marked by asterisk symbols show greatly weakened intensities, but still exist. The weakened intensities of Ni peaks indicate that after heating treatment, some of Ni NPs diffuse through thick carbon shells, and could be subsequently removed by chemical dealloying treatment.

Table S8. Summary of ORR mass activities reported in recent bimetallic PtNi-ORR literatures.

Reference	Catalyst	Electrolyte	Scan rate (mV/S)	Rotation (rpm)	MA (A/mg_{Pt})
Choi et al.	Octahedral	0.1 M $HClO_4$	10	1600	1.24

(2018)[11]	PtNi@Pt/C				
Cui et al. (2013)[12]	Activated PtNi octahedra	0.1 M $HClO_4$	5	1600	1.7
Li et al. (2016)[13]	J-PtNWs/C	0.1 M $HClO_4$	20	1600	13.6
Chen et al. (2014)[14]	Nanoframe Pt_3Ni/C	0.1 M $HClO_4$	20	1600	5.7
Choi et al. (2013)[15]	Octahedron $Pt_{2.5}Ni$/C	0.1 M $HClO_4$	10	1600	3.3
Cui et al. (2012)[16]	9.5 nm sized PtNi octahedra	0.1 M $HClO_4$	5	1600	1.45
Gan et al. (2013)[17]	Air–D–$PtNi_3$	0.1 M $HClO_4$	5	1600	0.75
Gan et al. (2013)[17]	N_2–D–$PtNi_3$	0.1 M $HClO_4$	5	1600	0.75
Gan et al. (2012)[18]	D–$PtNi_3$	0.1 M $HClO_4$	5	1600	0.81
Gan et al. (2012)[18]	D–PtNi	0.1 M $HClO_4$	5	1600	0.59
Gan et al. (2012)[18]	D–$PtNi_5$	0.1 M $HClO_4$	5	1600	0.58
Niu et al. (2016)[6b]	9 nm $Pt_{2.4}Ni$/C	0.1 M $HClO_4$	10	1600	2.67
Rudi et al. (2014)[19]	3.5 nm sized $PtNi_3$	0.1 M $HClO_4$	5	1600	0.55
Chattot et al. (2017)[20]	PtNi/C	0.1 M $HClO_4$	5	1600	0.246
Chattot et al. (2017)[20]	A-PtNi/C	0.1 M $HClO_4$	5	1600	0.26
Chattot et al. (2017)[20]	Ni@Pt/C	0.1 M $HClO_4$	5	1600	0.273
Chattot et al. (2017)[20]	Hollow PtNi/C	0.1 M $HClO_4$	5	1600	0.594

Chattot et al. (2017)[20]	Sea sponge PtNi/C	0.1 M $HClO_4$	5	1600	0.852
Beermann et al. (2017)[21]	PtNi_raw	0.1 M $HClO_4$	5	1600	1.7
Beermann et al. (2017)[21]	PtNi_300	0.1 M $HClO_4$	5	1600	2.7
Beermann et al. (2017)[21]	PtNi_500	0.1 M $HClO_4$	5	1600	0.7
Tian et al. (2019)[22]	PtNi-BNSs/C	0.1 M $HClO_4$	10	1600	1.89
Tian et al. (2019)[22]	PtNi-BNCs/C	0.1 M $HClO_4$	10	1600	3.52
Becknell et al. (2015)[23]	Pt_3Ni (1.0)	0.1 M $HClO_4$	20	1600	0.75[a]
Becknell et al. (2015)[23]	Pt_3Ni (1.5)	0.1 M $HClO_4$	20	1600	1.46[a]
Beermann et al. (2018)[24]	Pt-Ni/C (HAc)	0.1 M $HClO_4$	5	1600	0.43
Beermann et al. (2018)[24]	Pt-Ni/C (HAc_300)	0.1 M $HClO_4$	5	1600	0.52
Beermann et al. (2018)[24]	Pt-Ni/C (HAc_500)	0.1 M $HClO_4$	5	1600	0.32
Our work	3-PtNi/NiNC	0.1 M $HClO_4$	20	1600	0.96
Our work	5-PtNi/NiNC	0.1 M $HClO_4$	20	1600	2.59

[a] Specific activity in mA/cm^2, determined at 0.95 V_{RHE}.

We have compared the ORR performance with other reported PtNi bimetallic catalysts. As shown in Table S8, our catalysts in this present study, although not having the highest mass activity, still exhibit one of the highest reproducible ORR performances, which exceeds those of other existing PtNi bimetallic catalysts in terms of mass activities when normalized by the mass of Pt, validating the advantages of our strategy for fabricating high-performance Pt-based catalysts.

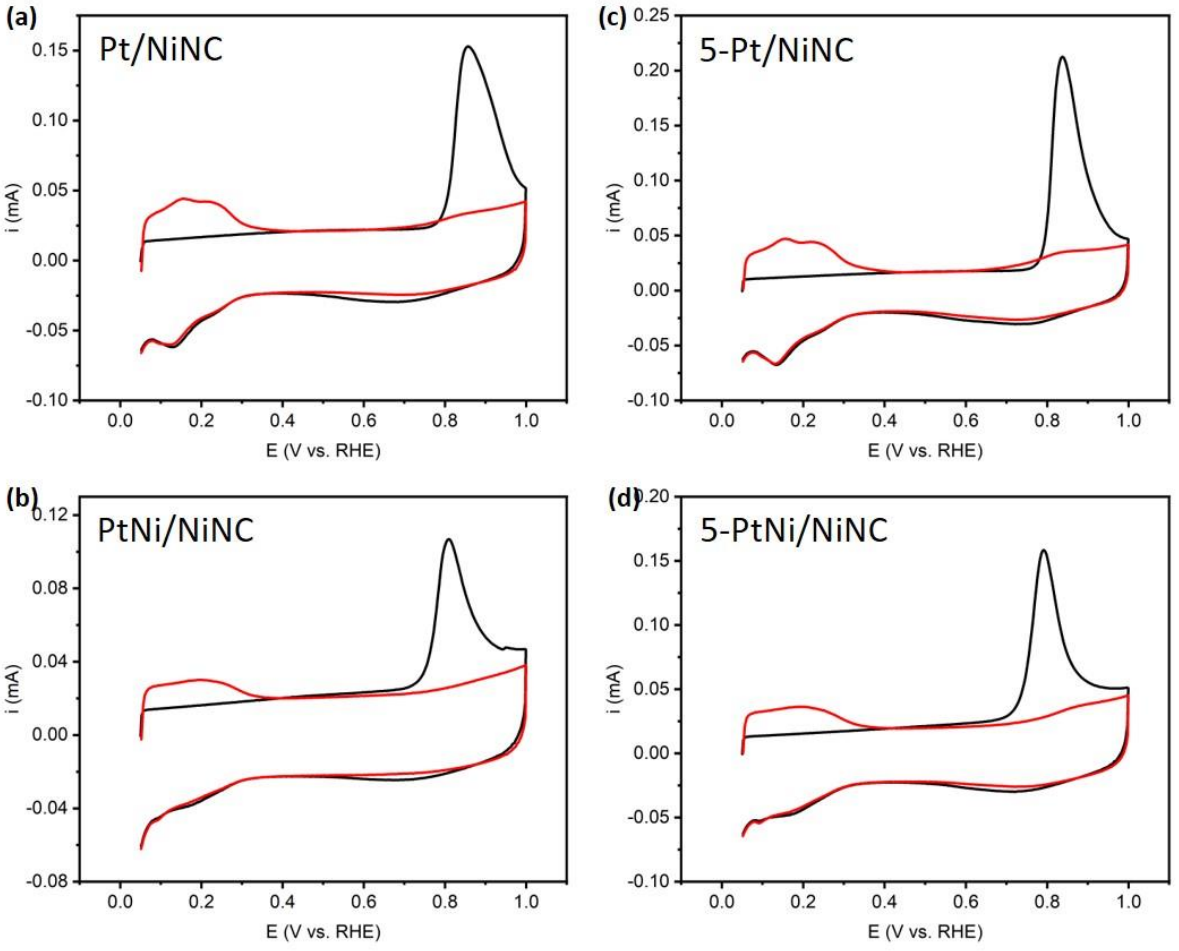

Figure S29. The CO stripping (black) and the following cyclic voltammetry (red) curves of our samples in a N_2-saturated 0.1 M $HClO_4$ solution. Scanning rate is 50 mV/s.

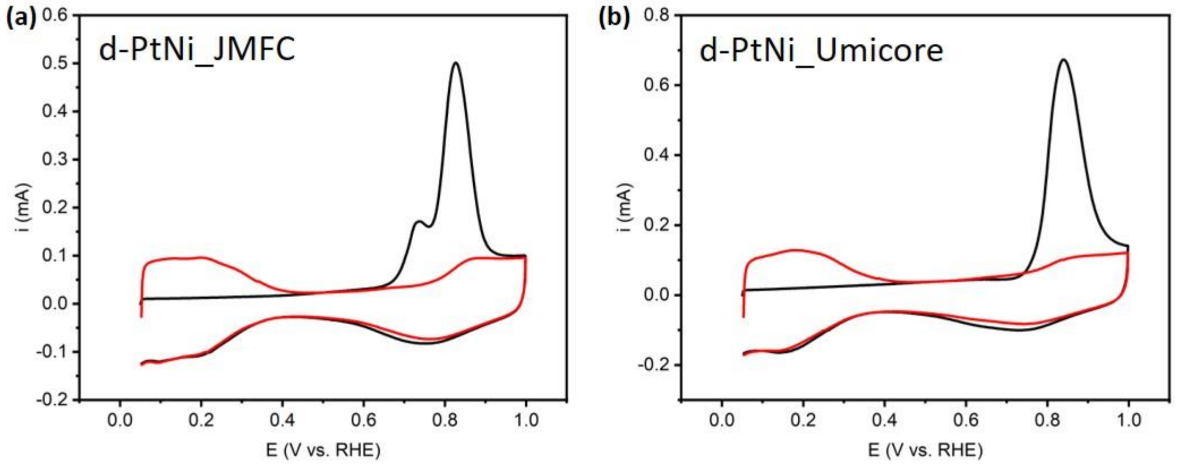

Figure S30. The CO stripping (black) and the following cyclic voltammetry (red) curves of two commercial PtNi catalysts in a N_2-saturated 0.1 M $HClO_4$ solution. Scanning rate is 50 mV/s. The CO-ECSA values of d-PtNi_JMFC and d-PtNi_Umicore are 63.7 ± 1.3 and 89.2 ± 6.6 m^2/g_{Pt}, respectively.

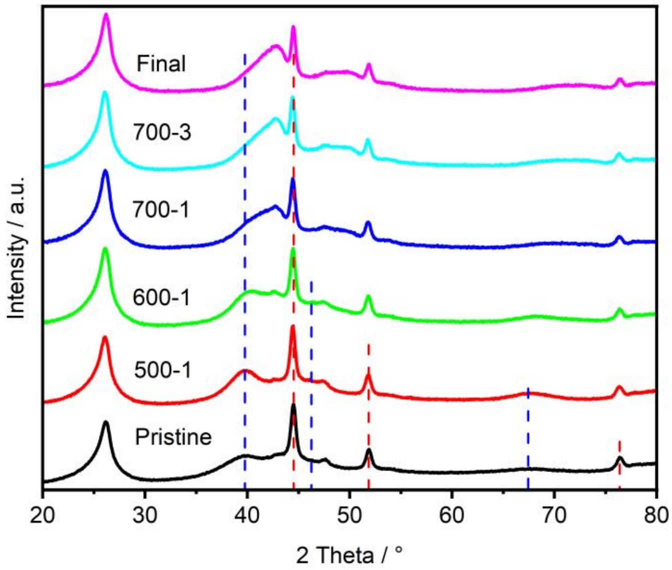

Figure S31. XRD pattern of pristine 5-Pt/NiNC and final annealed 5-PtNi/NiNC, together with four intermediate catalyst materials (500-1, 600-1, 700-1 and 700-3). The annealing condition for the final one is 700-6, that is 700 °C for 1 h.

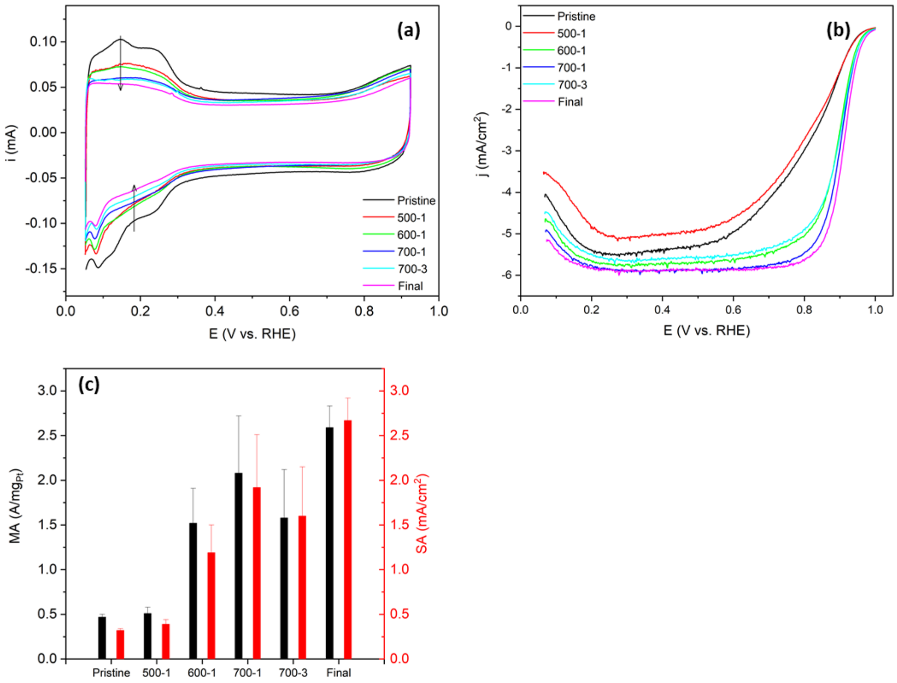

Figure S32. (a) CV curves, (b) LSV curves, and (c) comparison of MA and SA for pristine 5-Pt/NiNC and final annealed 5-PtNi/NiNC, together with four intermediate catalyst materials (500-1, 600-1, 700-1 and 700-3). The annealing condition for the final one is 700-6, that is 700 °C for 6 h.

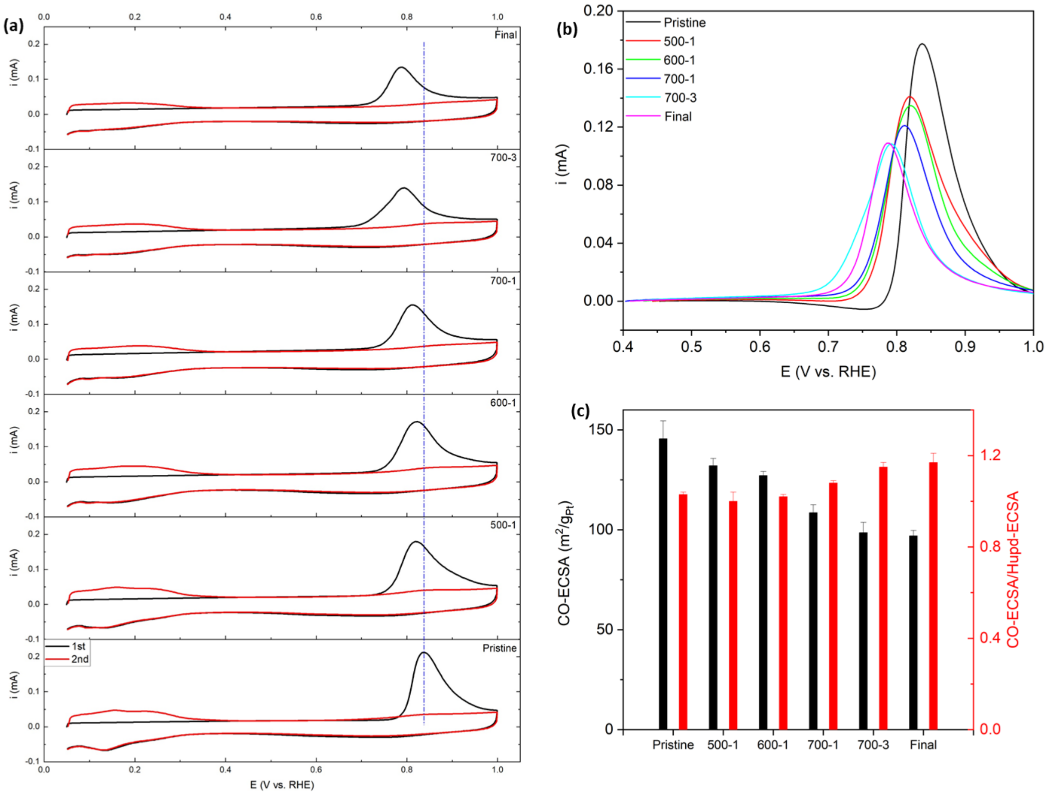

Figure S33. (a) Raw and (b) background-corrected CO stripping curves, and (c) comparison of CO-ECSA value and CO-ECSA/H_{upd}-ECSA ratio for pristine 5-Pt/NiNC and final annealed 5-PtNi/NiNC, together with four intermediate catalyst materials (500-1, 600-1, 700-1 and 700-3). The annealing condition for the final one is 700-6, that is 700 °C for 6 h.

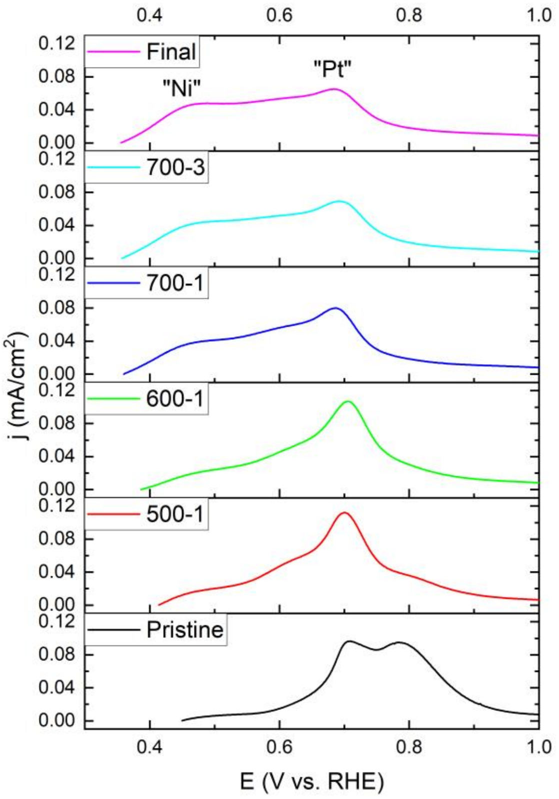

Figure S34. Pt surface area-normalized and background-subtracted CO stripping voltammetry in 0.1 M KOH solution for pristine 5-Pt/NiNC and final annealed 5-PtNi/NiNC, together with four intermediate catalyst materials (500-1, 600-1, 700-1 and 700-3). The annealing condition for the final one is 700-6, that is 700 °C for 6 h. Prior to CO stripping experiment in alkaline solution, these six samples underwent the same pretreatment in acidic solution (UL600acid-50C, 50 cycles between 0.05 V_{RHE} and 0.6 V_{RHE}).

Supporting Videos

Video S1

Image sequences of Pt/NiNC-7 catalyst during in situ heating TEM.

Video S2

Image sequences of Pt/NiNC-1 catalyst during in situ heating TEM.

References

[1] N. Masciocchi, F. Castelli, P. M. Forster, M. M. Tafoya, A. K. Cheetham, *Inorg. Chem.* **2003**, *42*, 6147–6152.
[2] a) S. Ott, A. Orfanidi, H. Schmies, B. Anke, H. N. Nong, J. Hübner, U. Gernert, M. Gliech, M. Lerch, P. Strasser, *Nat. Mater.* **2019**, *19*, 77–85; b) A. Orfanidi, P. Madkikar, H. A. El-Sayed, G. S. Harzer, T. Kratky, H. A. Gasteiger, *J. Electrochem. Soc.* **2017**, *164*, F418–F426.
[3] a) A. Zitolo, V. Goellner, V. Armel, M.-T. Sougrati, T. Mineva, L. Stievano, E. Fonda, F. Jaouen, *Nat. Mater.* **2015**, *14*, 937–942; b) A. Zitolo, N. Ranjbar-Sahraie, T. Mineva, J. Li, Q. Jia, S. Stamatin, G. F. Harrington, S. M. Lyth, P. Krtil, S. Mukerjee, E. Fonda, F. Jaouen, *Nat. Commun.* **2017**, *8*, 957.
[4] a) K. Kodama, T. Nagai, A. Kuwaki, R. Jinnouchi, Y. Morimoto, *Nat. Nanotechnol.* **2021**, *16*, 140–147; b) M. K. Debe, *Nature* **2012**, *486*, 43–51; c) L. Zhang, Y. Xia, *Adv. Mater.* **2014**, *26*, 2600–2606; d) G. Niu, A. Ruditskiy, M. Vara, Y. Xia, *Chem. Soc. Rev.* **2015**, *44*, 5806–5820; e) C. Roy, B. P. Knudsen, C. M. Pedersen, A. Velázquez-Palenzuela, L. H. Christensen, C. D. Damsgaard, I. E. L. Stephens, I. Chorkendorff, *ACS Catal.* **2018**, *8*, 2071–2080.
[5] H. Duan, D. Wang, Y. Li, *Chem. Soc. Rev.* **2015**, *44*, 5778–5792.
[6] a) L. Zhang, G. Niu, N. Lu, J. Wang, L. Tong, L. Wang, M. J. Kim, Y. Xia, *Nano Lett.* **2014**, *14*, 6626–6631; b) G. Niu, M. Zhou, X. Yang, J. Park, N. Lu, J. Wang, M. J. Kim, L. Wang, Y. Xia, *Nano Lett.* **2016**, *16*, 3850–3857.
[7] a) M. DeGraef, M. E. McHenry, *Structure of Materials: An Introduction to Crystallography, Diffraction, and Symmetry*, Cambrige University Press, Cambridge, **2007**; b) B. D. Cullity, S. R. Stock, *Elements of X ray diffraction*, 3 ed., Prentice Hall, New York, **2001**.
[8] a) F. Calle-Vallejo, J. I. Martínez, J. Rossmeisl, *Phys. Chem. Chem. Phys.* **2011**, *13*, 15639–15643; b) F. Luo, A. Roy, L. Silvioli, D. A. Cullen, A. Zitolo, M. T. Sougrati, I. C. Oguz, T. Mineva, D. Teschner, S. Wagner, J. Wen, F. Dionigi, U. I. Kramm, J. Rossmeisl, F. Jaouen, P. Strasser, *Nat. Mater.* **2020**, *19*, 1215–1223.
[9] V. Yarlagadda, M. K. Carpenter, T. E. Moylan, R. S. Kukreja, R. Koestner, W. Gu, L. Thompson, A. Kongkanand, *ACS Energy Lett.* **2018**, *3*, 618–621.
[10] S. Wei, A. Li, J.-C. Liu, Z. Li, W. Chen, Y. Gong, Q. Zhang, W.-C. Cheong, Y. Wang, L. Zheng, H. Xiao, C. Chen, D. Wang, Q. Peng, L. Gu, X. Han, J. Li, Y. Li, *Nat. Nanotechnol.* **2018**, *13*, 856–861.
[11] J. Choi, J.-H. Jang, C.-W. Roh, S. Yang, J. Kim, J. Lim, S. J. Yoo, H. Lee, *Appl. Catal. B: Environ.* **2018**, *225*, 530-537.
[12] C. Cui, L. Gan, M. Heggen, S. Rudi, P. Strasser, *Nat. Mater.* **2013**, *12*, 765–771.
[13] M. Li, Z. Zhao, T. Cheng, A. Fortunelli, C.-Y. Chen, R. Yu, Q. Zhang, L. Gu, B. V. Merinov, Z. Lin, E. Zhu, T. Yu, Q. Jia, J. Guo, L. Zhang, W. A. Goddard, Y. Huang, X. Duan, *Science* **2016**, *354*, 1414–1419.
[14] C. Chen, Y. Kang, Z. Huo, Z. Zhu, W. Huang, H. L. Xin, J. D. Snyder, D. Li, J. A. Herron, M. Mavrikakis, M. Chi, K. L. More, Y. Li, N. M. Markovic, G. A. Somorjai, P. Yang, V. R. Stamenkovic, *Science* **2014**, *343*, 1339–1343.
[15] S.-I. Choi, S. Xie, M. Shao, J. H. Odell, N. Lu, H.-C. Peng, L. Protsailo, S. Guerrero, J. Park, X. Xia, J. Wang, M. J. Kim, Y. Xia, *Nano Lett.* **2013**, *13*, 3420-3425.
[16] C. Cui, L. Gan, H.-H. Li, S.-H. Yu, M. Heggen, P. Strasser, *Nano Lett.* **2012**, *12*, 5885-5889.
[17] L. Gan, M. Heggen, R. O'Malley, B. Theobald, P. Strasser, *Nano Lett.* **2013**, *13*, 1131-1138.
[18] L. Gan, M. Heggen, S. Rudi, P. Strasser, *Nano Lett.* **2012**, *12*, 5423-5430.
[19] S. Rudi, C. Cui, L. Gan, P. Strasser, *Electrocatalysis* **2014**, *5*, 408–418.
[20] R. Chattot, T. Asset, P. Bordet, J. Drnec, L. Dubau, F. Maillard, *ACS Catal.* **2017**, *7*, 398-408.
[21] V. Beermann, M. Gocyla, S. Kühl, E. Padgett, H. Schmies, M. Goerlin, N. Erini, M. Shviro, M. Heggen, R. E. Dunin-Borkowski, D. A. Muller, P. Strasser, *J. Am. Chem. Soc.* **2017**, *139*, 16536–16547.
[22] X. Tian, X. Zhao, Y.-Q. Su, L. Wang, H. Wang, D. Dang, B. Chi, H. Liu, E. J. M. Hensen, X. W. Lou, B. Y. Xia, *Science* **2019**, *366*, 850-856.
[23] N. Becknell, Y. Kang, C. Chen, J. Resasco, N. Kornienko, J. Guo, N. M. Markovic, G. A. Somorjai, V. R. Stamenkovic, P. Yang, *J. Am. Chem. Soc.* **2015**, *137*, 15817–15824.
[24] V. Beermann, S. Kühl, P. Strasser, *J. Electrochem. Soc.* **2018**, *165*, J3026–J3030.

Author Contributions

Q.F. conceived and designed the study, analyzed data and wrote the manuscript. Q.F. carried out the material synthesis, characterizations and electrochemical measurements. X.W. performed the *in situ* heating TEM experiments. M.K. conducted the XPS measurements. M.H. performed the HRTEM, HAADF-STEM and STEM-EDX characterizations. P.S. supervised this study. All authors read and commented on the manuscript.
